# CRISPR/Cas9-mediated gene editing in trophoblast cells via mechanoporation for preeclampsia insight

**DOI:** 10.1038/s41419-025-08200-z

**Published:** 2025-11-24

**Authors:** Dorsa Morshedi Rad, Claire Richards, Sareh Zhand, Natasha de Alwis, Natalie J Hannan, Alen Faiz, Lana McClements, Majid Ebrahimi Warkiani

**Affiliations:** 1 School of Biomedical Engineering, Faculty of Engineering and Information Technology, University of Technology Sydney, Sydney, NSW 2007 Australia; 2 Institute for Biomedical Materials and Devices, Faculty of Science, University of Technology Sydney, Sydney, NSW Australia; 3 School of Life Sciences, Faculty of Science, University of Technology Sydney, Sydney, NSW Australia; 4 Therapeutics Discovery and Vascular Function in Pregnancy Group, Department of Obstetrics, Gynaecology and Newborn Health, The University of Melbourne, Melbourne, VIC Australia; 5 Respiratory Bioinformatics and Molecular Biology, School of Life Sciences, Faculty of Science, University of Technology Sydney, Sydney, NSW Australia

**Keywords:** Disease model, Cell delivery

## Abstract

Preeclampsia is a severe pregnancy complication marked by impaired trophoblast function and abnormal placental development, leading to significant maternal and fetal morbidity. FK506-binding protein-like (FKBPL) has been identified as a potential biomarker as it is significantly downregulated in early pregnancy stages of women who progress to develop preeclampsia. However, editing the *Fkbpl* gene in trophoblast cells to create a model of preeclampsia using clustered regularly interspaced short palindromic repeats (CRISPR)/CRISPR-associated protein 9 (Cas9) technology is challenging due to inefficient delivery, leading to low editing efficiency and reduced cell viability. To address these challenges, we developed a cost-effective and minimally invasive mechanoporation system using micro-engineered filters to deliver CRISPR/Cas9 plasmid DNA (pDNA) targeting the *Fkbpl* gene into trophoblast cells. This approach successfully generated cell lines with a 38% knockout (K/O) of *Fkbpl* expression, significantly reducing cell migration (wildtype (WT): 28.77% ± 4.7 vs. 38% K/O: 4.95% ± 0.8, wound closure, **p < 0.01) and proliferation (WT: 1.26 ± 0.06 vs. 38% K/O: 0.81 ± 0.01, ****p < 0.0001). Lower *Fkbpl-*K/O efficiency of 17% showed a similar reduction in cell proliferation as the 38% K/O clone. Although a full *Fkbpl*-K/O in the ACH-3P first-trimester trophoblast cell line was not achieved, the partial K/O provided valuable insights into *Fkbpl’s* role in trophoblast function relevant to preeclampsia pathogenesis. Moreover, treatment with mesenchymal stem cell (MSC)-derived small extracellular vesicles (sEVs) or MSC-sEVs did not restore migratory capacity in *Fkbpl*-deficient cells (*p* = 0.14). MSC-sEVs increased proliferation in WT ACH-3P cells at 1 µg (*p* < 0.05) and 2 µg (*p* < 0.01) doses, however, were not effective in either 17% or 38% *Fkbpl-*K/O clones, suggesting that FKBPL is an important mechanism of MSC-sEV-mediated therapeutic effect in trophoblasts in the context of preeclampsia. This study advances gene-editing techniques in placental biology and proposes new therapeutic strategies and mechanisms for pregnancy-related complications.

**Figure:**
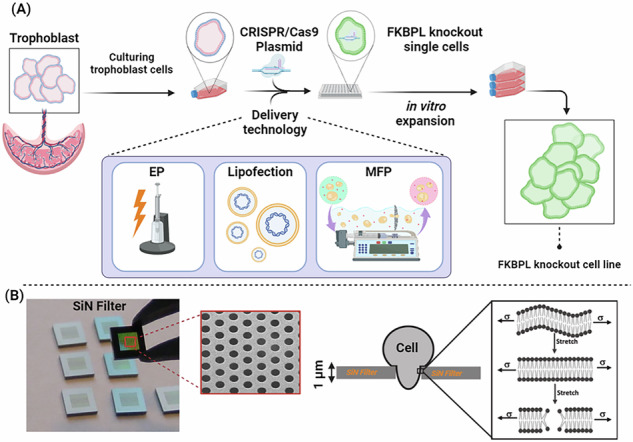
**A** Schematic overview of CRISPR/Cas9 plasmid delivery using microfiltroporation compared to gold standard electroporation and lipofection technologies in trophoblast cells. A CRISPR/Cas9 plasmid targeting *Fkbpl* was delivered to the first trimester trophoblast cell line, ACH-3P. Cells were sorted according to green fluorescence protein (GFP) expression, expanded and assessed for changes in cell function using proliferation and migration assays. **B** Actual images of the isopore silicon nitride (SiN) microfilters used in this study and diagram of cell membrane dynamics in response to mechanoporation. This figure was created with Biorender.com. CRISPR clustered regularly interspaced short palindromic repeats, EP electroporation, MFP microfiltroporation.

**A** Schematic overview of CRISPR/Cas9 plasmid delivery using microfiltroporation compared to gold standard electroporation and lipofection technologies in trophoblast cells. A CRISPR/Cas9 plasmid targeting *Fkbpl* was delivered to the first trimester trophoblast cell line, ACH-3P. Cells were sorted according to green fluorescence protein (GFP) expression, expanded and assessed for changes in cell function using proliferation and migration assays. **B** Actual images of the isopore silicon nitride (SiN) microfilters used in this study and diagram of cell membrane dynamics in response to mechanoporation. This figure was created with Biorender.com. CRISPR clustered regularly interspaced short palindromic repeats, EP electroporation, MFP microfiltroporation.

## Introduction

Hypertensive disorders of pregnancy, including preeclampsia, are a leading cause of maternal and neonatal morbidity and mortality. Preeclampsia is a condition that typically manifests after 20 weeks of gestation and affects 2–8% of pregnancies. Placental health relies on a tightly regulated angiogenic balance, with FKBPL being implicated as an important mechanism in placental dysfunction and preeclampsia pathogenesis [1, 2]. Early reductions in *Fkbpl* expression have been identified in women who later develop preeclampsia, suggesting a potential role for this gene in the pathogenesis of the disease [1].

Our recent research has revealed FKBPL and its target protein, cluster of differentiation 44 (CD44), as a new mechanism in the pathogenesis of preeclampsia [1]. *Fkbpl* is a peptidyl-prolyl cis-trans isomerase with potent anti-angiogenic activity. While highly expressed in placental tissue, its role in placental development and growth remains unclear. Complete homozygous knockout (K/O) of *Fkbpl* in mice resulted in embryonic lethality, while heterozygous K/O impaired vasculature [3]. In women before or with preeclampsia, FKBPL plasma concentration was initially significantly reduced but later significantly increased, respectively, suggesting a complex role in the disease [1]. Elucidating the role of *Fkbpl* in physiological pregnancy is crucial for developing innovative molecular interventions to address reproductive disorders and placental dysfunction.

The CRISPR/Cas9 technology, often referred to as “genetic scissors”, has been a groundbreaking tool for precise genome editing since its discovery in 2012 [4]. While CRISPR/Cas9 technology is a powerful gene-editing tool, it has limitations, including the potential for off-target effects and technical challenges in creating stable K/O cell lines, especially for essential genes like *Fkbpl*. *Fkbpl*’s role in cell survival, angiogenesis, and cell cycle regulation makes it particularly difficult to generate stable K/O cell lines, despite the potential benefits of such models for studying preeclampsia.

CRISPR/Cas9 systems can be delivered using carrier-based methods such as viral vectors, nanoparticles, or electroporation. While viral vectors offer high delivery efficiency, they face challenges including limited packaging capacity, high costs, safety concerns, delayed cell proliferation, and potential immunogenicity [5]. Nanoparticles and electroporation provide alternative delivery methods with potential advantages, but they also come with their own set of challenges, including stability, potential toxicity, and variability in delivery performance. Recent advancements in microengineering have led to the development of innovative membrane-disruption delivery methods. These include techniques such as cell squeezing (forcing cells through micron-sized channels smaller than the cell diameter), hydroporation (applying fluid shear stress to cells), and sonoporation (using ultrasound to create temporary pores in the cell membrane) [5–7]. While these technologies show promise for effectively bypassing the cell membrane barrier and delivering CRISPR/Cas9 systems into cells, their practical application in clinical settings is limited, especially for targeting hard-to-transfect and primary cells [5].

Microfilter-based mechanoporation (MFP) is an emerging non-viral, physical transfection strategy that addresses key limitations of conventional delivery methods such as electroporation and lipofection, including cytotoxicity, low reproducibility, and variable transfection efficiency. MFP operates by mechanically deforming cells as they are forced through microfabricated constrictions with diameters smaller than that of the cells, generating transient, size-controlled membrane disruptions. These disruptions facilitate the direct cytosolic entry of exogenous nucleic acids and other macromolecules, while preserving membrane integrity and ensuring high post-treatment viability [8]. Recently, we demonstrated that ultrathin silicon-based isopore filters can be effectively used for the delivery of large cargo into mammalian cells with high efficiency and minimal cytotoxicity [9]. Moreover, the MFP platform is amenable to integration with low-voltage electroporation, creating a hybrid modality that synergistically enhances transfection efficiency while preserving cell integrity. The physical nature of MFP circumvents many of the limitations associated with biochemical or electrical delivery systems, making it particularly advantageous for applications requiring high-throughput processing, reproducibility, and maintenance of cellular function [8–11].

In this study, we hypothesized that MFP could offer a novel and effective method for delivering CRISPR/Cas9 pDNA targeting *Fkbpl* in a first-trimester trophoblast cell line, ACH-3P. While traditional delivery methods such as electroporation and lipofection were tested, we aimed to explore an alternative approach that could enhance gene-editing efficiency and reduce associated drawbacks. Our method mimicked the reduced *Fkbpl* levels observed early in pregnancy in women who later developed preeclampsia [1, 12]. Given that *Fkbpl* plays a role in angiogenesis and inflammation, both of which are disrupted in preeclampsia, we explored whether MSC-sEVs, known for their regenerative and immunomodulatory properties, could help restore impaired cellular mechanisms resulting from *Fkbpl* disruption [3]. This approach highlights the novelty of our microfilter-based delivery system in advancing placental research and addressing reproductive disorders, as well as the therapeutic potential of MSC-sEVs and FKBPL-relevant mechanisms for managing preeclampsia-associated dysfunction.

## Materials and methods

### Placental tissue collection

Human placental samples were collected from a total of 36 pregnancies divided into three groups: 10 cases from the first trimester (gestational age range: 7–11 weeks), 16 cases from the preterm (gestational age range: 24–32 weeks), and 10 cases from the term stage (gestational age range: 37–39 weeks). For the collection of donated placental tissue, ethical approval was obtained from Mercy Health Human Research Ethics Committee (R11/34) and Austin Health Human Research Ethics Committee (HREC/18/Austin/44). Participants provided informed, written consent for sample collection. Experiments were performed following institutional guidelines and regulations. Pregnant women with chronic hypertension, renal disease, diabetes mellitus, preeclampsia, major fetal anomalies, infection, fever, or intrauterine fetal death were excluded from the study. Additional information on participant characteristics is provided in Supplementary Table 1.

First-trimester placental tissue was obtained from conceptus material collected at surgical terminations of singleton pregnancies (7–11 weeks of gestation) under general anesthesia via curettage, or both aspiration and curettage. Control healthy, term (37–39 weeks gestation) and preterm (24–32 weeks gestation) placentae were collected from normotensive pregnancies where a fetus of normal birth weight (>10th centile relative to gestation) was delivered. Placentae with evidence of chorioamnionitis, confirmed by placental histopathology, were excluded. All preterm and term patients gave birth or delivered via cesarean section. First-trimester placental tissue was identified and isolated from conceptus material, washed in phosphate-buffered saline (PBS; Thermo Fisher Scientific, United States) and transferred to cold DMEM Glutamax (Gibco, United Kingdom) until processing (within 4 h of collection). Term and preterm placental tissue were collected within 30 minutes of delivery, and four sites of the placenta were dissected (removing maternal and fetal surfaces) and washed in ice-cold PBS. All placental tissues were then preserved in RNAlater (Thermo Fisher Scientific, United States) for 48 h, after which the tissue was snap frozen and stored at −80 °C for subsequent analysis.

### Quantitative polymerase chain reaction of placental tissue

RNA was extracted from placental tissues using the GenElute Mammalian Total RNA Miniprep Kit (Sigma-Aldrich, Australia). The RNA was quantified using a Nanodrop 2000 spectrophotometer (Thermo Fisher Scientific, United States). Extracted RNA was converted to cDNA using the Applied Biosystems High-Capacity cDNA Reverse Transcription Kit, following the manufacturer’s instructions on the iCycler iQ5 (Bio-Rad, United States). Quantitative Taqman PCR was performed to quantify mRNA expression of *Fkbpl* (Hs01934566_s1; Life Technologies, United States) and reference gene cytochrome c1 (*Cyc1*) (Hs00357717_m1; Life Technologies, United States) on the CFX384 (Bio-Rad, United States) with the following run conditions: 95 °C for 20 s; 95 °C for 3 s, 60 °C for 30 s (40 cycles; Taqman Fast Advanced Master Mix). All data were normalized to the appropriate reference gene (stability confirmed) as an internal control. All cDNA samples were run in duplicate.

### Cell culture

The ACH-3P trophoblast cell line, developed by the fusion of primary first-trimester trophoblasts and choriocarcinoma cells (AC1-1), was kindly provided by Professor Gernot Desoye (Medical University of Graz, Austria). ACH-3P cells were maintained in Ham’s F12 culture medium (Thermo Fisher Scientific, United States) supplemented with 10% fetal bovine serum (FBS; Thermo Fisher Scientific, United States) and 1% penicillin-streptomycin (P/S; Thermo Fisher Scientific, United States). Every 2–5 passages, ACH-3P cells were treated with a selection medium containing azaserine (5.7 μM; Merck, Germany) and hypoxanthine (100 μM; Merck, Germany) to prevent choriocarcinoma overgrowth [13]. ACH-3P cells were used between passages 16–28. Primary bone marrow-derived mesenchymal stem cells (BM-MSCs; ATCC, United States) were initially expanded in ATCC mesenchymal stem cell basal medium and growth kit (ATCC, United States) prior to maintenance in Dulbecco’s Modified Eagle’s Medium (DMEM)/F12 + GlutaMAX (Thermo Fisher Scientific, United States) with 10% FBS and 1% P/S. BM-MSCs were used between passages 2–5. Both cell types were dissociated using StemPro Accutase (Sigma-Aldrich, United States) and were routinely tested for mycoplasma. Cells were incubated in a humidified atmosphere of 37 °C and 5% CO_2_.

### CRISPR/Cas9 pDNA design to knock out *FKBPL*

First, the single guide RNA (sgRNA) sequences targeting highly conserved DNA-binding domain regions of *Fkbpl* were designed and selected using a web-based resource, Benchling software (https://www.benchling.com/crispr/). Next, synthesized sgRNAs (Integrated DNA Technologies, United States) were cloned into the pSpCas9(BB)-2A-GFP (PX458), a gift from Feng Zhang (RRID:Addgene_48138, Addgene, United States), using the BbsI restriction site according to the previously published protocol [14]. To validate the integration of the sgRNA sequences into the pDNA, Sanger sequencing was performed (Australian Genome Research Facility (AGRF), Australia). Detailed information about the sgRNA and primer sequences is presented in Supplementary Table 2.

### CRISPR/Cas9 pDNA delivery to ACH-3P cells

Cargo delivery conditions were optimized by loading 70 kDa fluorescent isothiocyanate (FITC; Thermo Fisher Scientific, United States) dextran molecules into ACH-3P cells using benchtop electroporation (Neon transfection system, Thermo Fisher Scientific, United States) and our MFP delivery unit. To optimize the electroporation conditions, we tested multiple parameters, including voltage (1200–1500 V), electric pulse width (10–20 msec) and number of pulses [1–3]. These conditions were compared to unstained negative controls that had not been incubated with FITC and endocytosis controls that were incubated with FITC but not exposed to electroporation. For optimizing the MFP conditions, a concentration of 1–5 × 10^6^ ACH-3P cells ml^−1^ was resuspended with 4 µM FITC dextran and passed through the SiN membrane micropores (8 µm pore size) under a wide range of flow rates (0.5–3.0 ml min^−1^) as described previously [11]. This range was selected to balance efficient processing with maintaining high cell viability. The proposed setup can handle a maximum flow rate of up to 5 ml min^−1^, as tested in optimization studies. However, while the system can technically accommodate these higher flow rates, the viability of the cells may be compromised, potentially decreasing at flow rates around or above this level. Cell viability and FITC fluorescence signal intensity were simultaneously evaluated by co-staining with SYTOX Blue dead cell stain (Thermo Fisher Scientific, United States). A 1:1000 dilution of the SYTOX Blue viability dye was added to the delivery buffer under conditions sensitive to light. Subsequent flow cytometry analysis and data processing were performed using the BD FACS LSR Fortessa cell analyzer and BD FACSDiva software (BD Biosciences, United States), respectively. Emissions at specified wavelengths for SYTOX Blue (excitation/emission: 444/480 nm) and FITC dextran molecules (excitation/emission: 495/521 nm) were measured to assess cell viability and delivery efficiency, respectively. Next, 5 µg of CRISPR/Cas9 pDNA targeting *Fkbpl* was introduced into 70-80% confluent ACH-3P cells (a stage chosen to optimize transfection efficiency while maintaining healthy cell proliferation) using either lipofection (Lipofectamine P3000, Thermo Fisher Scientific, United States), electroporation or SiN-based MFP under the optimized conditions. Upon transfection, ACH-3P cells were incubated in serum-free Ham’s F12 culture medium at 37 °C with 5% CO_2_.

### Fluorescent activated cell sorting (FACS) of transfected cells and clonal expansion

After 24 h following transfection, ACH-3P cells were detached and resuspended in FACS buffer containing PBS and 5% FBS. The cell suspensions were passed through the Falcon 40 µm cell strainer (Sigma-Aldrich, United States) to achieve a homogenous suspension of single cells. Next, ACH-3P cells were single-cell sorted based on GFP fluorescence using a BD FACSMelody cell sorter (BD Biosciences, United States) into 96-wells. ACH-3P cells were identified based on forward vs. side scatter area (FCS-A vs. SSC-A), and GFP fluorescence signals were identified using a blue laser (excitation 488 nm, emission 530/30 nm). The baseline fluorescence level was defined using non-transfected (negative control) ACH-3P cells. The single-cell sorted ACH-3P cells were then maintained at 37 °C with 5% CO_2_ for 2–3 weeks for clonal expansion and downstream analysis. All data were captured for the first 10,000 events detected and analyzed using the BD FACS Chorus software (BD Biosciences, United States).

### Quantification of FKBPL expression in CRISPR/Cas9-transfected cells

Once the ACH-3P clones reached sufficient confluency, the cells were washed with PBS three times, and 500 µL of RIPA lysis buffer (50 mM Tris-HCl, 150 mM NaCl, 0.1% Triton X-100, 0.5% sodium deoxycholate, 0.1% SDS, pH = 8) containing 1% Halt protease inhibitor cocktail (Thermo Fisher Scientific, United States) was added to lyse the cells. The cell lysate was incubated on ice for 30 min before centrifugation for 10 min at 14,000 rpm and 4 °C. Protein supernatant was collected and stored at −80 °C. Protein was quantified using a Pierce BCA Protein Assay kit (Thermo Fisher Scientific, United States), and 10 µg of the sample was reduced with 4× Bolt LDS Sample Buffer (Thermo Fisher Scientific, United States) in a 1:4 dilution and incubated at 70 °C for 10 min. Samples were run alongside a 2:3 protein standard combination (MagicMark and Benchmark Novex; Thermo Fisher Scientific, United States) in 4–12% Bis-Tris gels (Thermo Fisher Scientific, United States) and transferred to PVDF membranes. Membranes were blocked with 5% skim milk buffer and probed with 1:1000 mouse anti-FKBPL monoclonal antibody (Abcam, United Kingdom), and 1:10,000 rabbit anti-GAPDH (Abcam, United Kingdom) in 5% skim milk overnight at 4 °C. Membranes were washed and incubated with their corresponding HRP-conjugated secondary antibody before washing. Membranes were visualized using Clarity Western ECL Substrate (Bio-Rad, United States) and Amersham Imager 600 (GE Healthcare Life Sciences, United States). Band pixel intensity was measured using ImageJ, normalized to GAPDH, and expressed as a fold change of the control.

### DNA extraction and Sanger sequencing

Genomic DNA was extracted from approximately 1 × 10⁶ wildtype (WT) and CRISPR K/O cells using the QIAamp DNA Mini Kit (Qiagen, Germany) according to the manufacturer’s protocol. DNA concentration and purity were assessed using a NanoDrop™ One Microvolume UV-Vis Spectrophotometer (Thermo Fisher Scientific, United States). The CRISPR-Cas9 target region was amplified by PCR using MyTaq DNA polymerase and reaction buffer (Bioline/Meridian Bioscience, United States) under the following thermal cycling conditions: initial denaturation at 94 °C for 2 min; 35 cycles of denaturation at 94 °C for 10 s, annealing at primer-specific temperature for 30 seconds, and extension at 68 °C for 2 min; followed by a final extension at 68 °C for 7 min and cooling at 4 °C.

PCR products were verified by gel electrophoresis on a 2% agarose gel (run at 130 V for 1 h). Amplicons were purified using the Invisorb Fragment Clean-up Kit (Invitek Molecular, Germany). For Sanger sequencing, 15 ng of purified PCR product per 100 bp was combined with 25 pmol of either forward or reverse sequencing primer (Supplementary Table 2) in a total volume of 20 µL of Milli-Q water. Sequencing was performed by the Australian Genome Research Facility (AGRF, Australia). Sanger sequencing data were analyzed using Synthego’s Inference of CRISPR Edits (ICE) tool (https://ice.synthego.com/).

### Isolation of sEVs from BM-MSCs

Human BM-MSCs (passage 4) were maintained in DMEM/F12 with 10% FBS and 1% P/S to reach ~80% confluency and incubated at 37 °C incubator with 5% CO_2_. Then, for conditioned medium preparation, the media was changed to DMEM/F12 and 1% P/S and the cells were incubated in a hypoxic chamber (37 °C, 5% CO_2_, and 2% O_2_) for 48 h to induce oxygen and serum deprivation. MSC-conditioned medium (MSC-CM) was then collected and used to isolate the sEVs according to the standard ultracentrifugation method [15]. Briefly, the MSC-CM was spun down in three steps as follows: (i) 300×*g* at 4 °C for 10 min, (ii) 2000×*g* at 4 °C for 20 min, and (iii) 10,000×*g* at 4 °C for 30 min using the R18A rotor of the high-speed refrigerated centrifuge (Hitachi, Japan) to remove cell and protein debris. An additional ultracentrifugation step at 100,000×*g* at 4 °C for 120 min was performed (Beckman Coulter, United States) to exclude the residual proteins in the MSC-sEV samples, followed by resuspending the MSC-sEV pellet in 100 µl of sterile DPBS that was filtered through a 0.22 µm syringe filter (Sigma-Aldrich, United States) and stored at −80 °C for downstream analysis.

### Characterization of MSC-derived sEVs

Upon MSC-sEVs quantification, characterization of the isolated sEVs was performed using nanoparticle tracking analysis (NTA), western blotting and scanning electron microscopy (SEM) analysis. The concentration and size distribution of the MSC-sEVs were identified by NTA using the NanoSight LM14 system (Malvern Panalytical, United Kingdom). The setting parameters were kept constant across samples with a threshold of 6 and slider shutter and gain of 650 and 50, respectively. The resulting data were then analyzed using NTA software version 3.3 (Malvern Panalytical, United Kingdom).

For western blotting, isolated sEVs were lysed using RIPA buffer (Thermo Fisher Scientific, United States), and their protein content was quantified using the Pierce BCA Protein Assay kit (Thermo Fisher Scientific, United States), according to the manufacturer’s protocol. 5 µg (equivalent to 2 × 10^8^ sEVs) of sEV protein were resolved using Bolt 4–12% Bis-Tris Plus Gels (Invitrogen, United States) and transferred to the PVDF membrane (Thermo Fisher Scientific, United States) alongside a combination of MagicMark and PageRuler Plus prestained protein ladder (Thermo Fisher Scientific, United States). Membranes were blocked for one hour at room temperature with 5% non-fat powdered milk in PBS-T (PBS and 0.5% Tween-20) and incubated overnight at 4 °C with primary antibodies against classical sEV markers, anti-human CD9, CD63, and CD81 (BioLegend, United States), and anti-CD44 (Abcam, United States) at 1:500 dilution in PBS-T, followed by incubation with HRP-conjugated Goat anti-mouse IgG as a secondary antibody (1:2000 in PBS-T). Then SuperSignalWest Dura Extended Duration Substrate (Thermo Fisher Scientific, United States) was used to visualize the blot. For SEM analysis, isolated sEVs were immobilized on 10 µm polystyrene beads functionalized with an anti-CD9 antibody. The SEM sample preparation was performed according to our previously published protocol [16]. Next, a 20 nm Au/Pd coating in a vacuum was used for sample sputter coating, and SEM images were captured using Zeiss Supra 55VP SEM with an accelerating voltage of 15 kV.

### Labeling and internalization of MSC-derived sEVs

The PKH67 fluorescent cell linker (Sigma-Aldrich, United States) was used to label the sEVs according to the manufacturer’s datasheet. Briefly, 100 µl of the MSC-sEV sample was added to the 3 µl of PKH67 dye in 1 mL Diluent C (Sigma-Aldrich, United States) and incubated for 10 min. The dye was quenched with 10% BSA and removed by ultracentrifugation at 190,000×*g* for 2 h. The stained sEVs were resuspended in PBS. PKH67-stained MSC-sEVs were added to WT or *Fkbpl*-K/O ACH-3P cells in a time-dependent manner (2, 4, and 6 h). sEVs that were not bound or internalized were removed by washing with PBS three times, and nuclei were labeled with Hoescht 33342 (Thermo Fisher Scientific, United States). MSC-sEV internalization was visualized using a Stellaris confocal microscope (Leica Microsystems, Germany) with a HC PL APO CS2 40× water immersion objective (NA 1.10), Lattice SIM microscope (Zeiss, Germany) equipped with a Hamamatsu Orca Flash 4.0 camera (C15440-20UP-50) or Nikon A1R confocal fluorescence microscope with Plan Apo λ 100× oil immersion objective (NA 1.45). Image analysis was performed using ImageJ (v1.53J).

### Scratch-wound assay

A scratch-wound assay was conducted to evaluate the migration of the CRISPR-edited ACH-3P cells in vitro. WT and *Fkbpl*-K/O cells were seeded in wells of a 96-well plate at a concentration of 16,000 cells per well. Cells were incubated in serum-reduced medium (as described above) for 10 h prior to treatment. Upon reaching confluency, cells were treated with medium containing PBS (16%) or MSC-sEV (0.25 µg well^−1^) for 24 h prior to wounding. A gentle cross scratching using a 96-pin WoundMaker tool (Essen BioScience, Ann Arbor, United States) was applied to the plate. The wells were washed twice with PBS to remove cell debris, and medium with PBS or MSC-sEVs was reapplied to each corresponding well. The plates were incubated in an IncuCyte live cell imaging system (Essen BioScience, United States) and two 10× objective images were acquired from each well every 2 h, for a total of 36 h. Images were analyzed using ImageJ to measure the wound area and calculate the percentage of wound closure over time. The experiments were performed in triplicate, and the results were expressed as the mean percentage of wound closure ± standard error of the mean.

### MTT assay

An MTT assay was performed using Thiazolyl Blue Tetrazolium Bromide dye (Sigma-Aldrich, United States). The WT ACH-3P and *Fkbpl*-K/O cells were seeded at a concentration of 10,000 cells per well in triplicate wells of a 96-well plate. Cells were serum-starved in serum-reduced medium (Ham’s F12 containing 1% FBS, 1% P/S) for 16 h. Cells were then incubated in 150 µl medium spiked with PBS (16%) or MSC-sEV (0.25, 1 or 2 µg well^−1^) for 24 h prior to the addition of MTT dye. Next, 15 µl of MTT reagent was added to each well, and the plates were incubated for 2 h at 37 °C. The MTT reagent was then removed, and the resulting formazan crystals were solubilized in 150 µl of Dimethylsulfoxide (DMSO; Sigma-Aldrich, United States). The plates were shaken for 10 min to ensure complete solubilization of the crystals. Absorbance was measured at 570 nm using the Tecan Infinite M Plex plate reader (Tecan Life Sciences, Switzerland). Absorbance at 650 nm was recorded as a background reading, and a well containing DMSO was used as a blank.

### Statistical analysis

The statistical analysis was performed via a two-tailed paired *t*-test and one-way analysis of variance (ANOVA). The one-way ANOVA was used to compare the means of more than two groups and assess whether there was a significant difference between the means of multiple experimental groups. Where data were normally distributed, a Tukey’s post hoc test was applied, while non-normally distributed data were assessed by Kruskal–Wallis post hoc test using GraphPad Prism 9.5.1 software. A threshold value of *p* value <0.05 was used to indicate statistical significance.

## Results

### Placental *Fkbpl* expression increases across gestation

**Fig. 1:**
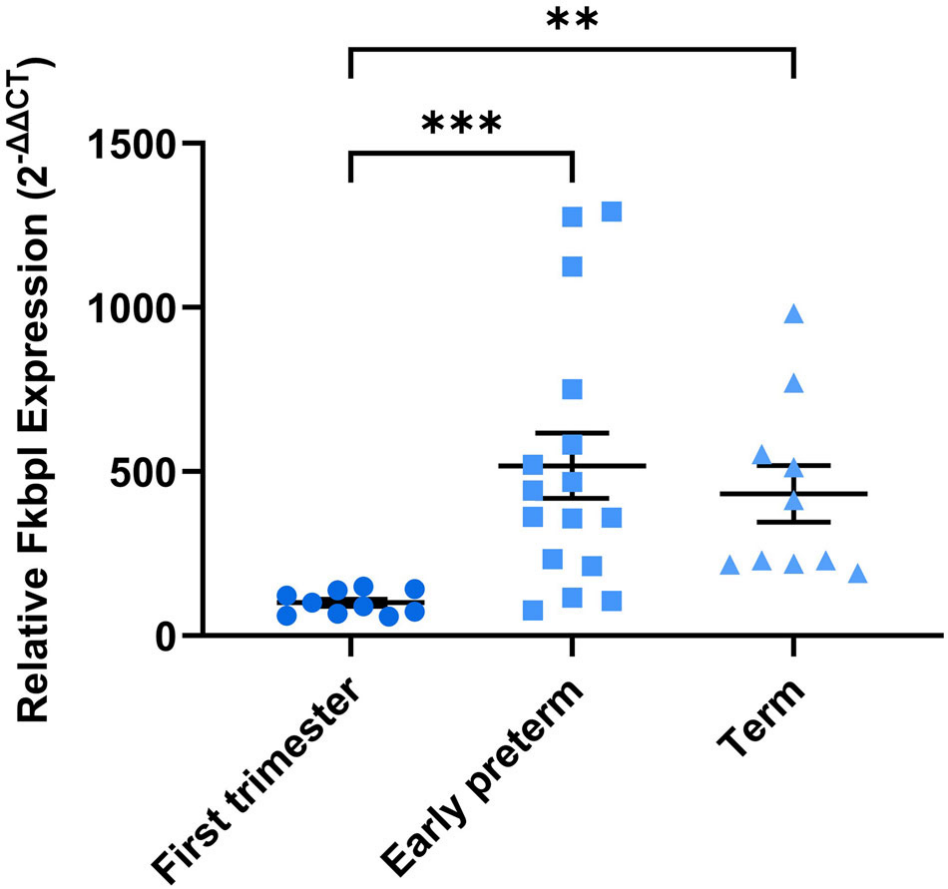
*Fkbpl* mRNA expression in placentae from different stages of pregnancy, including first trimester, preterm, and term. Biopsies from first trimester (7–11 weeks), early preterm (24–32 weeks) and term (≥37 weeks) placentae were collected, and mRNA extracted. *Fkbpl* expression was assessed by qPCR and normalized to Cyc1. Data were not normally distributed according to the Shapiro–Wilk normality test, so was analyzed by ordinary one-way ANOVA with Kruskal–Wallis post hoc test; plotted as mean ± SEM; *n* ≥ 10; **p < 0.01, ***p < 0.001.

To characterize *Fkbpl* expression in physiological pregnancies across the gestation period, *Fkbpl* mRNA was assessed in human placental samples. In this study, 36 human placental samples were collected from three different stages of pregnancy: first trimester (7.4–11.0 weeks, *n* = 10), preterm (24.3–32.1, *n* = 16), and term (37–39 weeks, *n* = 10). Clinical characteristics of the participants are summarized in Supplementary Table 1. Systolic (sBP) and diastolic blood pressure (dBP) measurements were reported only for preterm and term cases, with no significant differences observed. To investigate whether *Fkbpl* plays a role in the observed changes during pregnancy, we evaluated the expression of *Fkbpl* at the mRNA level in placental samples. To check that gestational age and sample collection times did not impact results, RNA quality was assessed by a bioanalyser, and all samples were confirmed to have an RNA integrity number (RIN) of 8–9, sufficient for PCR analysis. The results indicated that *Fkbpl* was expressed at low levels during the first trimester; however, this significantly increased during the early preterm period (Fig. 1). *Fkbpl* was then maintained, with no significant difference between the preterm and term samples.

### MFP delivers FITC dye at high efficiency with improved cell survival

As we were introducing CRISPR/Cas9 pDNA into ACH-3P trophoblast cells for the first time, optimization was performed for both the electroporation and MFP systems. Our primary aim was to successfully load a 70 kDa FITC dextran molecule into the ACH-3P cells using both electroporation and MFP as delivery methods. Given the lack of electroporation data available for this cell line, we tested different parameters such as voltage (1200–1500 V), pulse width (10, 15, and 20 msec) and the number of electric pulses (1–3×). This voltage range was selected to systematically explore conditions that would enable effective electroporation while minimizing cellular damage. Since ACH-3P cells had not been previously electroporated, our objective was to identify the optimal parameters to ensure maximum transfection efficiency and cell viability. By testing a voltage range of 1200–1500 V, we aimed to determine the threshold that would be sufficient to permeabilize the cell membrane without causing excessive stress or cell death [17]. Furthermore, to optimize the MFP delivery conditions, we forced ACH-3P cells through SiN microfilters at various flow rates between 0.5 to 2.0 ml min^−1^, which was in line with our previously published research [11]. This allowed us to identify the optimal flow rate that would facilitate the efficient delivery of exogenous cargo molecules inside ACH-3P cells.

**Fig. 2:**
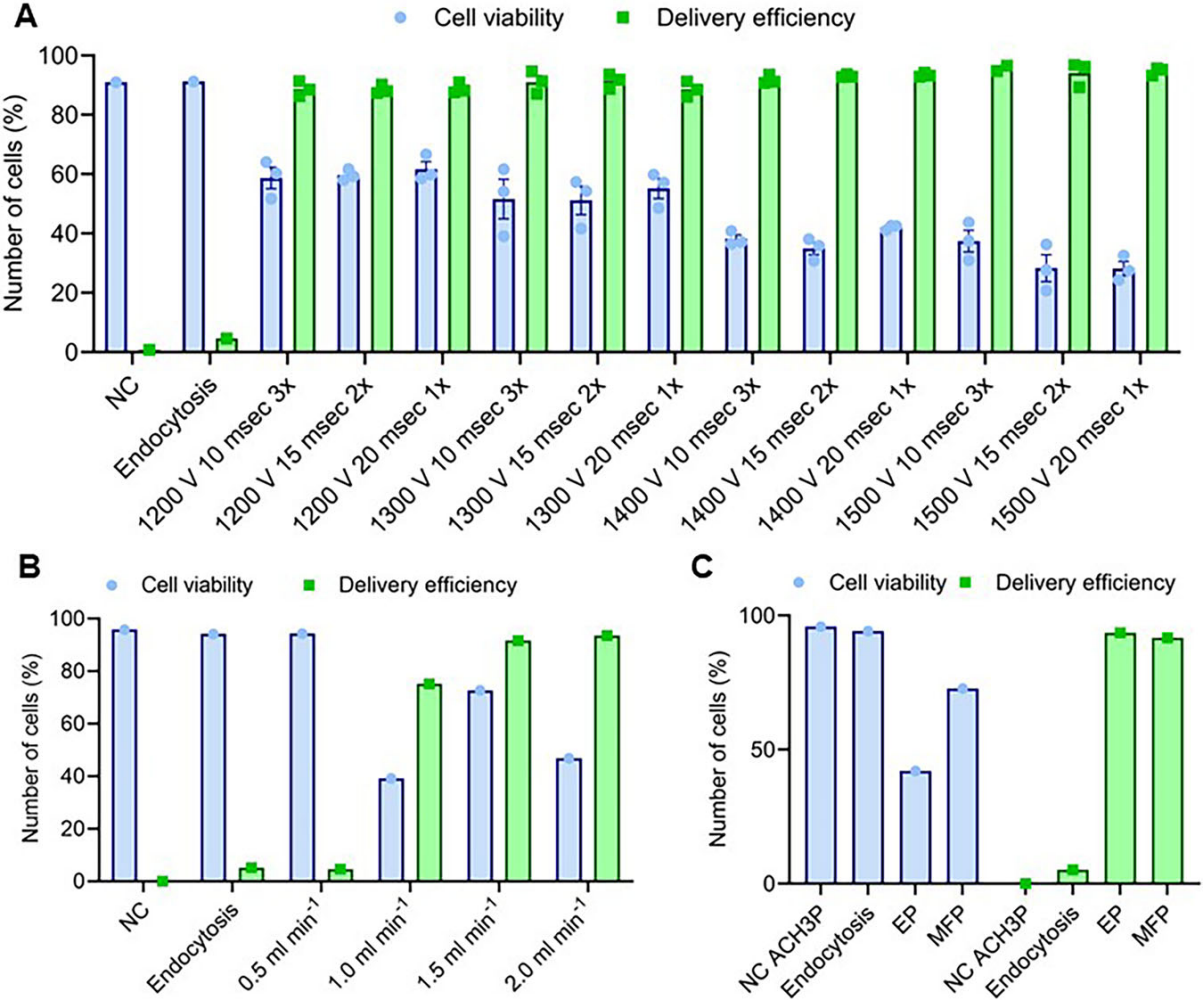
Optimization of electroporation and microfiltroporation by assessing delivery of 70 kDa FITC dextran dye into ACH-3P cells. Cell viability was assessed by 444/480 nm and FITC uptake measured by 495/521 nm laser by flow cytometry. ACH-3P cell viability and delivery efficiency following **A** electroporation and **B** microfiltroporation under different conditions. **C** Direct comparison of optimal electroporation and microfiltroporation parameters. Data expressed as individual points or mean ± SEM; *n* = 1-3. NC non-treated control, EP electroporation, MFP microfiltroporation.

Our study revealed that increasing the intensities of electric pulses, including voltage, pulse width, and the number of electric pulses, led to a reduction in cell viability in the electroporation samples (Fig. 2A). The viability of cells in the electroporation group ranged from 28.1 to 61.7%, with an average of 46.7%. However, the delivery efficiency in all tested conditions exceeded 80%, except for cells subjected to 1500 V, 10 msec, 3×. We identified the optimal electroporation conditions to achieve the highest delivery efficiency while maintaining acceptable cell viability. The most effective delivery conditions were achieved at 1400 V for 20 msec and 1×, which resulted in 93.5% delivery efficiency and 42.0% cell viability, suggesting that ACH-3P cells are inherently sensitive to electric shock.

To determine the optimal delivery conditions for the MFP setup, we passed a mixture of ACH-3P cells and 70 kDa FITC dextran molecules through SiN microfilters at different flow rates (0.5–2.0 ml min^−1^). In our optimization experiments, microporous filters with an 8 µm pore size were used. This pore size was specifically chosen as it approximately corresponds to 50% deformation of the average cell diameter, which balances effective membrane disruption and has minimal impact on cell functionality. By selecting this pore size, we aimed to ensure sufficient permeabilization while minimizing excessive mechanical stress or deformation that could adversely affect cell viability and function. Our findings revealed that cell viability decreased sharply at a flow rate of 1.0 ml min^−1^, increased at 1.5 ml min^−1^, and then declined again at the flow rate of 2 ml min^−1^ (Fig. 2B). Therefore, we established the optimal delivery conditions for the MFP setup at 1.5 ml min^−1^, as this flow rate resulted in the best delivery outcomes, with 91.7% of cells loaded with 70 kDa FITC dextran while maintaining a viability rate of 72.8%. Next, we compared the optimal delivery performance of the MFP setup with electroporation (Fig. 2C). Although there was no difference in delivery efficiency, ACH-3P cells subjected to the MFP system maintained higher cell viability. This suggests that MFP can be a safe and highly efficient delivery option for transfecting ACH-3P cells.

### MFP delivers CRISPR/Cas9 pDNA with greater cell survival

**Fig. 3:**
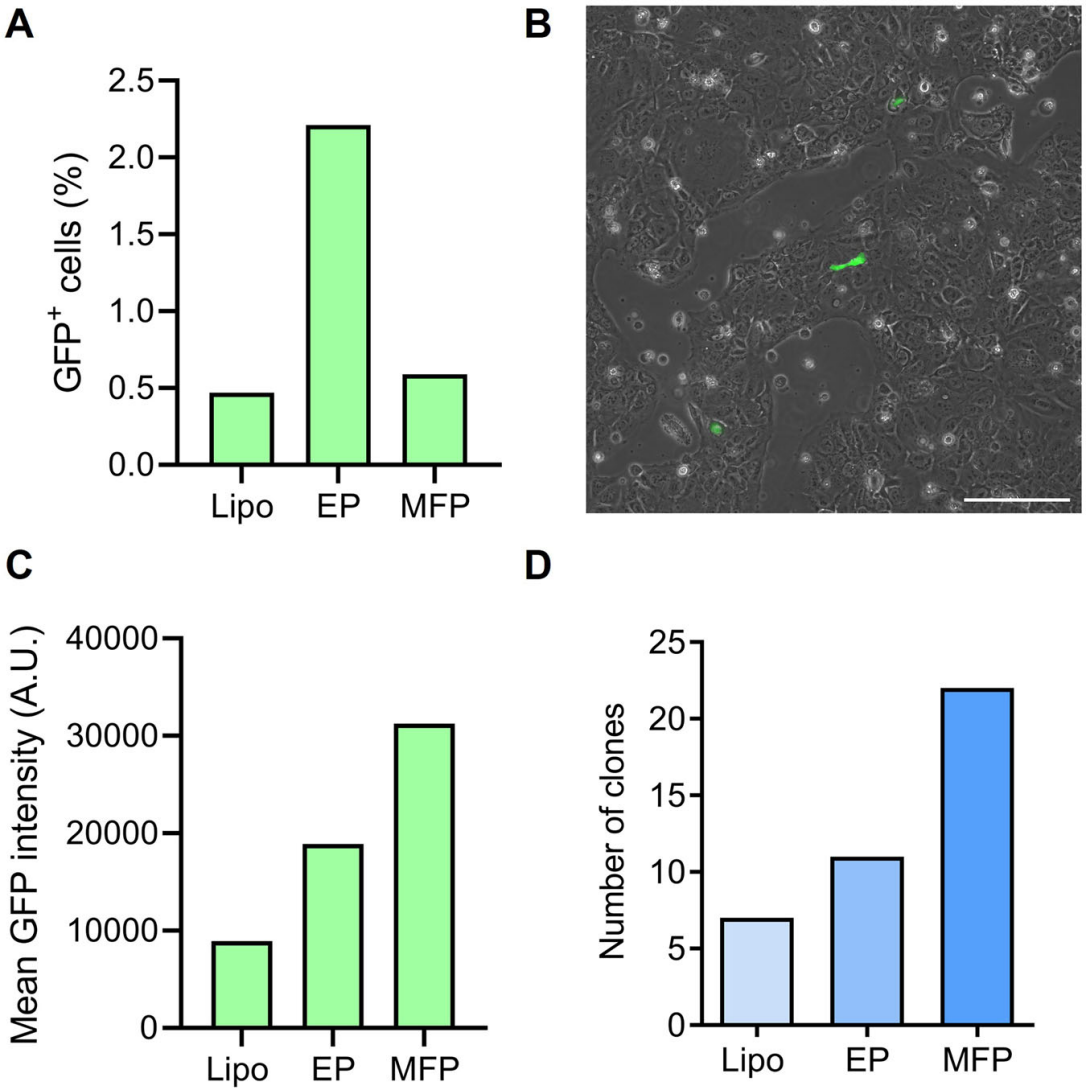
Green fluorescent protein (GFP) expression and number of surviving clones following *Fkbpl*-targeting CRISPR/Cas9 pDNA delivery into trophoblasts. **A** Percentage of GFP-expressing cells of 10,000 CRISPR/Cas9-transfected cells per delivery method. **B** Representative image of GFP-expressing ACH-3P cells treated with MFP, acquired using EVOS FL microscope at 10× magnification, scale bar = 200 µm. **C** Mean intensity of GFP in CRISPR/Cas9 transfected cells. **D** Number of surviving clones following single-cell sorting of GFP^+^ CRISPR/Cas9-transfected cells. *N* = 1. Lipo lipofection, EP electroporation, MFP microfiltroporation.

To determine the optimal method of delivering a CRISPR/Cas9 pDNA targeting *Fkbpl*, we employed MFP and compared transfection outcomes with two benchtop technologies, lipofection and electroporation. To maintain consistency throughout the experiments, the cells and CRISPR/Cas9 pDNA concentrations were kept constant. The application of electroporation to the ACH-3P trophoblast cells led to a notable 57.14% increase in the number of GFP^+^ cells (Fig. 3A), though the MFP-transfected cells exhibited higher GFP signal intensities (Fig. 3B), suggesting greater efficiency in delivering pDNA compared to the benchtop technologies (Fig. 3C). Further, although the number of GFP^+^ cells was lower in the MFP-transfected group compared to the electroporation-transfected group, the number of surviving clones that were capable of expansion was greater in the MFP group (Fig. 3D). This indicates that our MFP technology may induce less cell damage during transfection. Western blotting analysis of edited clones using lipofection or electroporation revealed variations in FKBPL protein expression across clones (Supplementary Fig. 1A, B). Some clones maintained FKBPL protein expression at levels comparable to negative controls, while some clones demonstrated reductions in FKBPL expression of as much as 76.6%. Although these experiments were not repeated, the data is based on clonal populations derived from single cells, which inherently reduces variability and strengthens the validity of the findings. Based on these results, MFP transfection method was chosen for downstream experiments.

Compared to lipofection and electroporation, MFP exhibits a significant advantage in preserving cellular viability following transfection, while still facilitating sufficient plasmid uptake to enable effective genome editing. Although electroporation resulted in a higher proportion of GFP⁺ cells (Fig. 3A), this was accompanied by a notable reduction in the number of viable clones capable of expansion (Fig. 3D), indicative of increased cellular stress or membrane-associated damage. Lipofection demonstrated both lower transfection efficiency and reduced post-transfection viability (Fig. 3A, D). In contrast, MFP achieved an optimal balance between delivery efficiency and cell survival, a feature that is particularly advantageous for downstream applications such as clonal selection, long-term propagation, and functional studies in sensitive cell types such as primary cells.

Our results demonstrated that the MFP delivery platform was highly effective in transfecting the ACH-3P cells with the Fkbpl-targeting CRISPR/Cas9 pDNA, yielding a two-fold increase in the number of surviving clones generated compared to the benchtop electroporation (Fig. 3D). The protein levels of FKBPL were assessed by Western Blotting for each of the expanded clones from MFP (Supplementary Fig. 1C). FKBPL expression again varied across clones, with some showing reductions of 79% (K/O6) and one sample with absent FKBPL protein expression (K/O23).

**Fig. 4:**
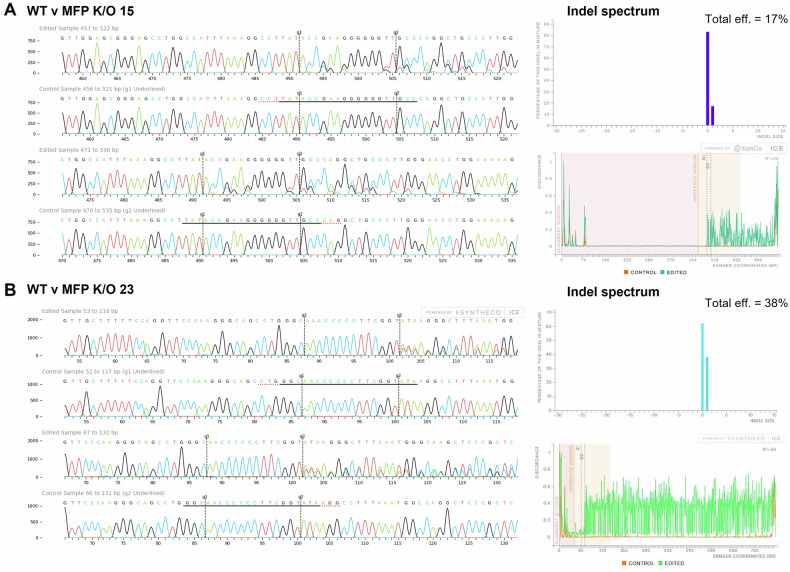
Sanger sequencing results of WT compared to edited cells through CRISPR/Cas9 delivery by microfiltroporation for K/O15 and K/O23 of *Fkbpl* gene regions. *Fkbpl* gene regions were amplified, and Sanger sequenced by AGRF. Sequences from **A** K/O15 and **B** K/O23 were compared to a WT control sequence using Synthego’s Inference of CRISPR edits (ICE) analysis tool (https://ice.synthego.com/). Indel insertion or deletion of a base pair.

DNA sequences of the lowest-expressing clones from each transfection approach were assessed following Sanger sequencing using Synthego’s ICE tool (Fig. 4A, B). Due to variations in *Fkbpl* expression in clones across the delivery technologies, we chose two clones with different K/O efficiencies of *Fkbpl* DNA sequences, which were MFP clone’s K/O15 and K/O23, with an indel of 17% and 38%, respectively. These results confirmed that we were unable to generate a complete *Fkbpl*-K/O cell line in ACH-3P cells and may signify the importance of *Fkbpl* in cell survival and clonal expansion.

### MSC-derived sEVs are internalized by trophoblasts

To further explore the potential impact of MSC-derived sEVs on trophoblast cell proliferation and migration and the role that *Fkbpl* has in this process, we initially isolated sEVs from MSCs. MSC-sEV characterization was performed through three critical methods: SEM, NTA, and western blotting for sEV markers including CD63, CD81, CD9, and CD44 (Supplementary Fig. 2). SEM images of polystyrene beads with a surface coating of MSC-derived sEVs confirmed the presence of sEVs in the isolated sample (Supplementary Fig. 2A, B). Furthermore, NTA results confirms that MSC-derived sEVs had an average particle size of 126 nm ± 56.2 nm (mean ± standard error) with a total concentration of 3.2 × 10^9^ particles ml^−1^ (Supplementary Fig. 2C). The western blotting results also confirmed the expression of three common sEV-specific markers in the isolated sample, further validating the presence of sEVs in the sample obtained from MSCs (Supplementary Fig. 2D).

**Fig. 5:**
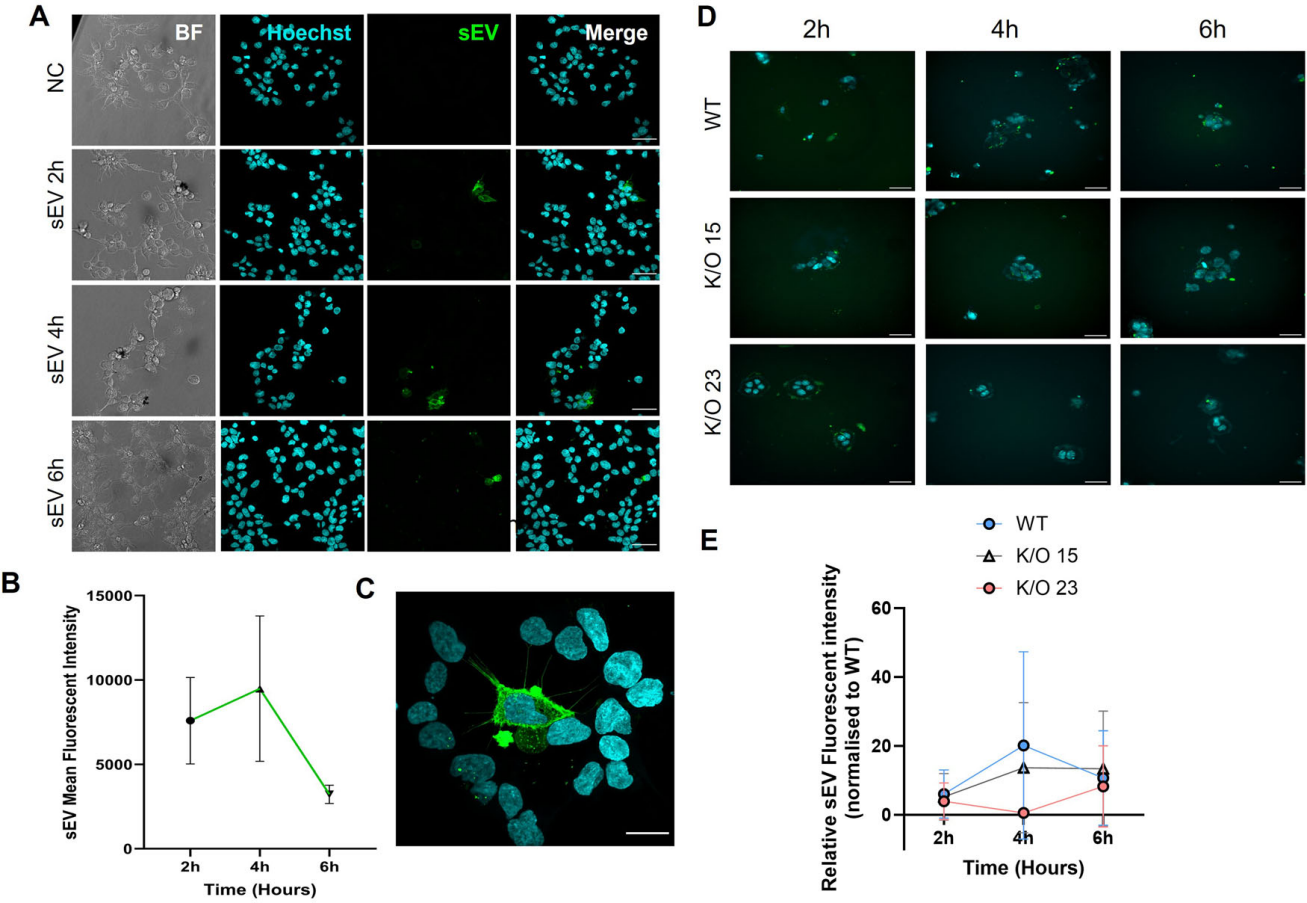
sEVs internalization within WT and *Fkbpl*-K/O ACH-3P trophoblast cells. sEVs were isolated from MSCs and labeled with PKH67 prior to incubation with ACH-3P cells. Cells were incubated for 2, 4 and 6 h prior to washing and fixing. Untreated cells were used as a negative control (NC). Hoechst 33342 was used to label nuclei. **A** Representative maximum intensity projection images. Images were acquired at 20× magnification using a Leica Stellaris confocal microscope. Scale bar = 50 µm. **B** Fluorescent intensity of PKH67-labeled sEVs internalized within trophoblast cells across incubation timepoints. Data expressed as mean ± SEM, *n* = 3. **C** High-resolution image acquired at 100× magnification using a Nikon A1R confocal microscope. The scale bar indicates 20 µm. **D** WT, K/O15 and K/O23 cells were treated with PKH67-labeled sEVs for 2, 4, and 6 h. Images were acquired with a Zeiss Lattice SIM microscope. Scale bar = 50 µm. **E** Mean fluorescent intensity of sEVs in cells was analyzed using ImageJ and normalized to the WT control. NC negative control, WT wildtype, K/O knockout, sEVs small extracellular vesicles.

**Fig. 6:**
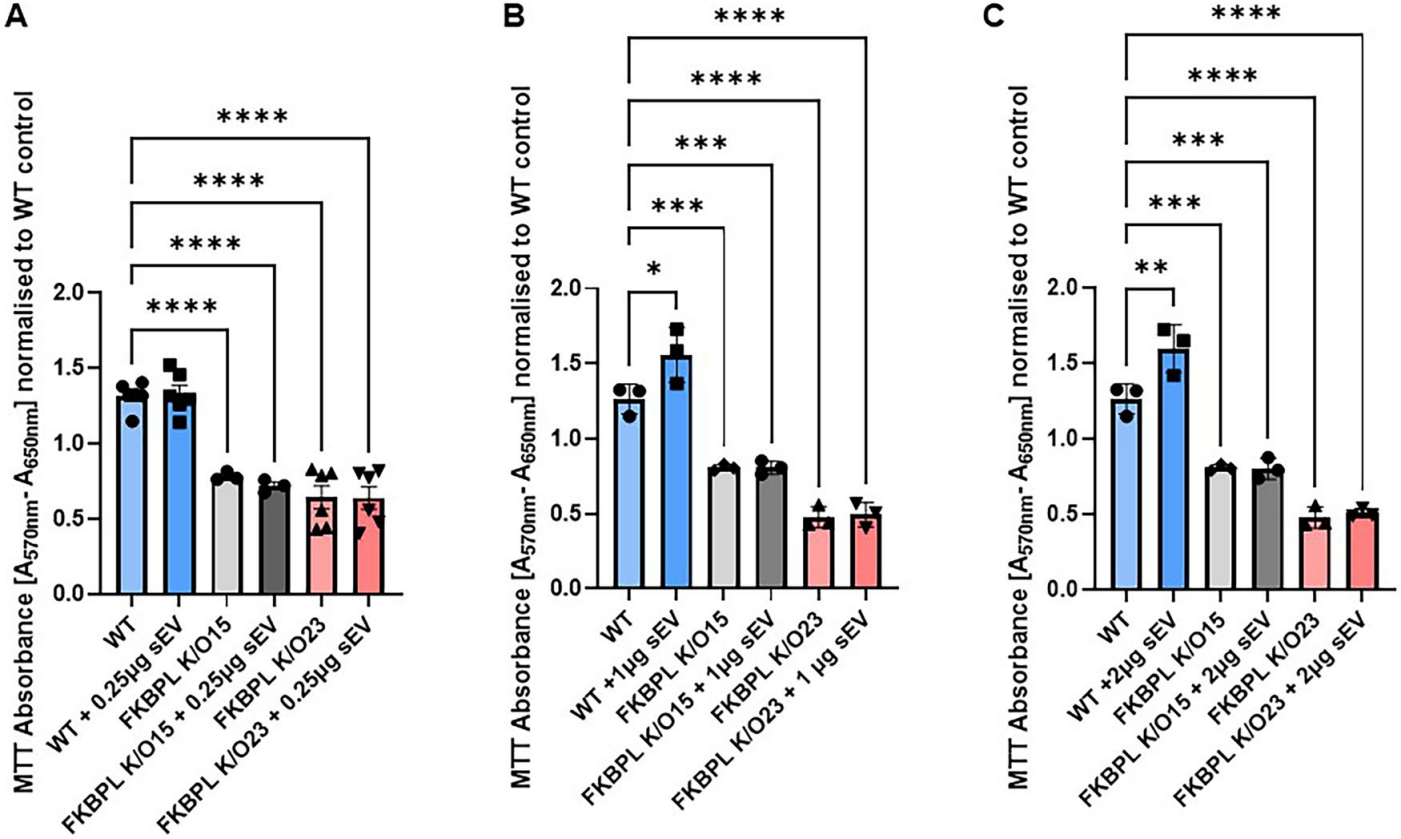
*Fkbpl* partial K/O reduces trophoblast cell proliferation that MSC-sEVs cannot restore. WT ACH-3P cells and *Fkbpl*-K/O15 and K/O23 clones treated with MSC-derived sEVs at **A** 0.25 µg, **B** 1 µg, and **C** 2 µg. Absorbance of MTT assay recorded at 570 nm with background subtraction of absorbance at 650 nm and normalised to WT control. Data were analyzed by one-way ANOVA with Sidak’s post hoc and are presented as mean ± SEM; *n* = 3-6; *p < 0.05; **p < 0.01, ***p < 0.001; ***p < 0.0001. sEVs small extracellular vesicles, K/O knockout, WT wildtype.

Following the characterization of MSC-derived sEVs, we confirmed the internalization of sEVs by trophoblast cells using confocal fluorescence microscopy. Our microscopy results indicated sEV internalization by trophoblasts had peaked by 4 h and an optimal incubation period of 6 h would ensure adequate uptake. As demonstrated in Fig. 5A, B, sEVs were successfully internalized by trophoblast cells within this timeframe. The cytoplasmic localization of fluorescently labeled sEVs was noted and the reduced intensity observed by 6 h may indicate trafficking to lysosomes for degradation or fusion with the endosomal membrane for content release. Interestingly, when we compared sEV internalization by WT vs K/O15 or K/O23 ACH-3P clones, lower sEV mean fluorescence intensity was noted for *Fkbpl*-K/O clones, irrespective of the K/O efficiency, which then increased at 6 h, suggesting a delayed sEV uptake and content release due to lower FKBPL expression (Fig. 6).

### *Fkbpl*-K/O impairs proliferation and migration in first-trimester trophoblast cells, with MSC-sEVs offering partial rescue

In this study, we evaluated the impact of *Fkbpl*-K/O on cell proliferation and migration in first-trimester trophoblast cells and explored the potential of MSC-derived sEVs to mitigate these effects. Figure 6A–C illustrates the impact of *Fkbpl* reduction on metabolic activity and proliferation, as assessed by an MTT assay. The reduction of *Fkbpl* significantly decreased both parameters compared to WT controls (WT 1.26 ± 0.06 vs. *Fkbpl*-K/O23 0.64 ± 0.08, vs *Fkbpl-*K/O15 0.78 ± 0.02 *p* < 0.0001), irrespective of the K/O efficiency. MSC-sEVs treatment at three doses tested (0.25 µg, Fig. 6A; 1 µg, Fig. 6B or 2 µg, Fig. 6C) did not rescue the impaired proliferation in the *Fkbpl*-K/O cells (*p* > 0.05), irrespectively of K/O efficiency. However, MSC-sEVs at 1 µg (Fig. 6B, *p* < 0.05) and 2 µg (Fig. 6C, *P* < 0.01) increased the proliferation of WT ACH-3P cells. These findings suggest that partial *Fkbpl*-K/O impairs trophoblast proliferation significantly, which can lead to inappropriate placental development. While MSC-sEVs improve trophoblast proliferation in WT ACH-3P, partial *Fkbpl-*K/O seems to impede their effect, independent of the percentage of *Fkbpl* reduction, potentially aligning with the delayed or reduced EV internalization observed in K/O clones.

**Fig. 7:**
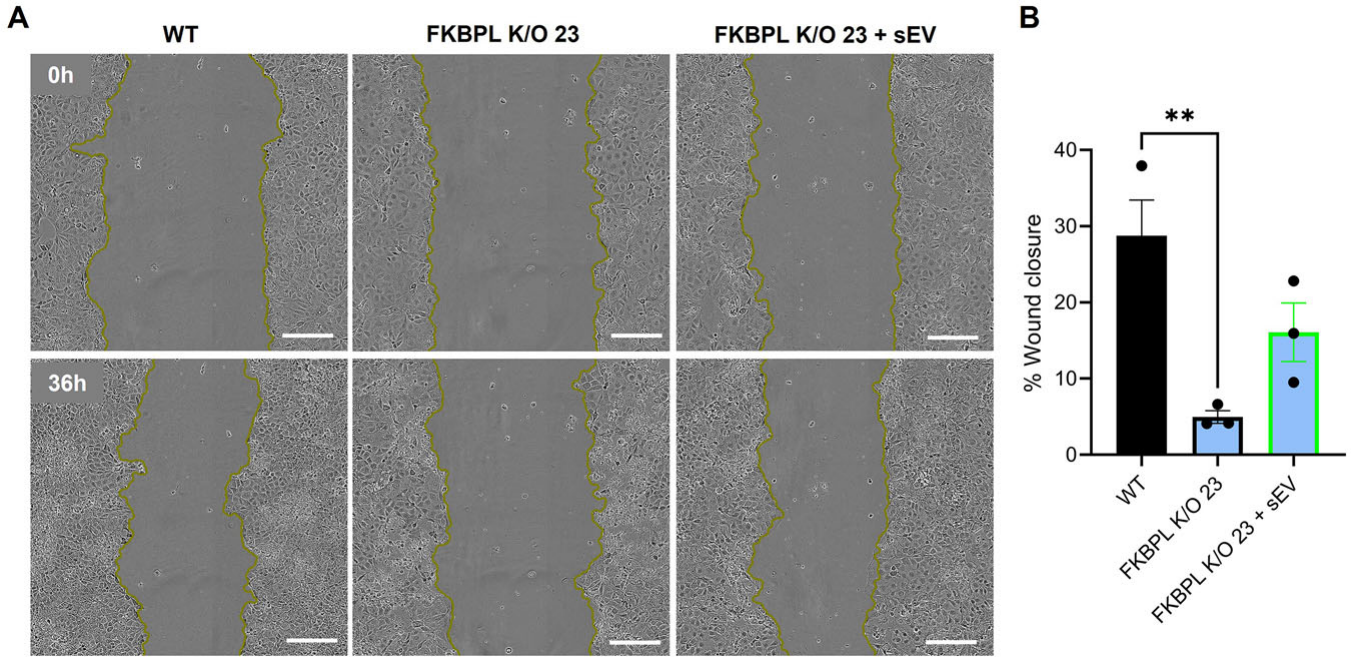
*Fkbpl* partial K/O impairs trophoblast migration. **A** Images of WT ACH-3P cells and *Fkbpl*-K/O23 clone treated with MSC-derived sEVs at *t* = 0 h and *t* = 36 h of the wound generation. The scale bar indicates 250 µm. **B** Percentage area of wound closure from 0 h to 36 h. Data were analyzed by one-way ANOVA with Tukey's post-hoc test and are presented as mean ± SEM; *n* = 3; **p < 0.01, ***p < 0.001. sEVs small extracellular vesicles, K/O knockout, WT wildtype.

Following on from the proliferation assay, Fig. 7A, B shows the results from a scratch-wound assay, demonstrating that *Fkbpl* is crucial for cell migration. The *Fkbpl*-K/O23 clone healed only 5% of the wound after 36 h, compared to 28% healing in WT cells (WT 28.77% ± 4.7 vs. *Fkbpl*-K/O 4.95% ± 0.8, **p = 0.007). MSC-sEVs treatment did not significantly improve migration in the *Fkbpl*-K/O23 clone (*Fkbpl*-K/O 4.95% ± 0.8 vs. *Fkbpl*-K/O + EVs 16.09% ± 3.8, *p* = 0.14).

## Discussion

In this study, we investigated the role of *Fkbpl* in ACH-3P placental cells under conditions relevant to preeclampsia, a significant pregnancy complication. Our research began by characterizing *Fkbpl* expression throughout physiological pregnancies and assessing its potential role in pregnancy-related changes. Our findings indicate that *Fkbpl* expression increases with gestational age, which correlates with placental growth. While our study did not examine the localization of *Fkbpl* in specific regions of the placenta, data from the Human Protein Atlas suggests that *Fkbpl* mRNA expression varies across trophoblast subtypes (https://www.proteinatlas.org/ENSG00000204315-FKBPL/single+cell/) potentially explaining the altered expression patterns observed during gestation. The variability in *Fkbpl* mRNA expression may also be influenced by differences in sample processing times, which is a limitation of our study. Nonetheless, the significant changes in *Fkbpl* expression observed during placental development and growth, along with its potential implications in preeclampsia, underscore the need for further research to understand the function of this gene in placental cells.

To address this gap, we employed CRISPR/Cas9-mediated gene-editing using MFP and electroporation for the first time to specifically evaluate the role of *Fkbpl* in ACH-3P trophoblast cells. Both techniques were optimized for payload delivery in ACH-3P cells. The results demonstrated that while both electroporation and MFP can effectively deliver molecules into ACH-3P cells, they have different impacts on cell viability. The observed decrease in cell viability with increasing voltage and pulse duration during electroporation suggests that higher intensities cause more significant membrane damage, increased cytotoxicity, and disruption of cellular processes. This trade-off between delivery efficiency and cell viability is a common challenge in electroporation. In contrast, the fluctuations in cell viability observed with varying MFP flow rates likely reflect a balance between mechanical stress and the efficiency of cell processing through the micropores. At lower flow rates, cells experience prolonged exposure to shear forces, which increases stress and reduces viability. Higher flow rates reduce exposure time but may increase turbulence or pressure, further stressing the cells. The optimal flow rate of 1.5 ml min^−1^ for MFP minimizes shear stress while avoiding excessive turbulence, resulting in improved cell viability. The comparison between MFP and electroporation reveals that while both methods can achieve high delivery efficiency, MFP offers the advantage of higher cell viability, suggesting that MFP is a safer and highly efficient option for transfecting ACH-3P cells. Despite these demonstrated benefits, MFP has not yet seen widespread adoption in the context of genome engineering. A principal barrier remains the requirement for the microfabrication of SiN microfilters, which are typically produced using microelectromechanical systems (MEMS)-based techniques in cleanroom environments. This dependence on specialized infrastructure limits accessibility for most biological laboratories. Furthermore, the absence of standardized, optimized protocols across diverse cell types and molecular cargos continues to hinder experimental reproducibility and broader applicability. To facilitate the integration of MFP into routine molecular and cellular workflows, future developments should prioritize alternative microfilter manufacturing strategies that are scalable and compatible with high-throughput applications, such as polymer-based or lithography-free fabrication techniques. In addition, the establishment of robust, cell-specific protocols and the incorporation of MFP platforms into modular, automated systems will be essential to enhance usability. Expanding the validation of MFP in a broader range of biologically relevant systems, including primary human cells, stem cells, and disease models, will further substantiate its potential as a valuable tool for genome editing and regenerative medicine research.

To identify the optimal transfection system for delivering *Fkbpl*-targeting CRISPR/Cas9 pDNA, we compared MFP with two benchtop technologies, electroporation and lipofection. Our results showed that while MFP-transfected cells exhibited a median number of GFP^+^ cells, they had higher GFP signal intensities compared to electroporated cells. The higher GFP signal intensities observed in MFP-transfected cells suggest that MFP may deliver a higher number of plasmids to a smaller subset of cells, resulting in more intense GFP expression. This finding indicates that MFP, although resulting in a lower initial number of GFP^+^ cells, may induce less cellular stress, preserving cell viability and enhancing recovery and expansion post-transfection. This balance between transfection efficiency and cell viability is critical in applications where long-term cell survival and proliferation are important, such as gene editing and therapeutic development. Since MFP generated more surviving K/O clones than electroporation and lipofection, this technology appears to be a promising option for transfecting trophoblast cells with CRISPR/Cas9 pDNAs.

The inability to create a complete *Fkbpl*-knockout cell line in ACH-3P cells may indicate the importance of *Fkbpl* gene in cell survival and clonal expansion. This finding aligns with previous evidence demonstrating that a homozygous knockout of *Fkbpl* at the organism level is embryonically lethal, which could imply that *Fkbpl*-K/O might also be lethal at the cellular level in trophoblasts [3]. Further experiments, such as a CRISPR screening library and validation in primary trophoblast cells, are necessary to confirm this hypothesis. Additionally, the natural variation in *Fkbpl* expression observed across surviving clones, despite genetic stability, may result from differences in expression across trophoblast subtypes within the ACH-3P cells. The ACH-3P cell line has been shown to contain a heterogeneous population of human leukocyte antigen-G (HLA-G) positive and negative cells, indicative of at least two different trophoblast subtypes [13]. Interestingly, small *Fkbpl-*K/O efficiencies (i.e., 17% and 38%) led to a larger reduction at the protein level (50% and 100%). This is not surprising given FKBPL is a chaperone protein that forms a complex with HSP90 [18] and E3 ligase, RBCK1 [19], which post-translationally regulate FKBPL expression.

Nevertheless, we showed here that irrespective of *Fkbpl*-K/O efficiencies, the cell phenotypes and response to treatment were similar, suggesting that even small changes in *Fkbpl* have substantial impact on cell function. Given that FKBPL is a chaperone protein which forms complexes with HSP90 [18] or CD44 [20], regulating multiple pathways downstream, compensatory pathways are likely to be involved following *Fkbpl-*K/O. Our previous work shows that FKBPL siRNA-mediated knockdown increases endothelial cell migration, hypoxia-inducible factor 1α and CD31 expression while inhibiting VE-cadherin [21]. In our study, the impact of partial *Fkbpl-*K/O was different on trophoblast cell proliferation and migration, suggesting cell-specific effects. In the context of trophoblast cells, reduced *Fkbpl* expression inhibited their critical functions in placental development which could lead to complications like preeclampsia. Compensatory mechanisms are likely to be involved following *Fkbpl-*K/O as we have previously described with FKBPL-based therapeutic peptide mimetic, AD-01. Some of these compensatory mechanisms in the context of cardiovascular disease involve tissue remodeling proteins, including collagen alpha-1 (XIX) chain and junctional cadherin-associated-5 (JCAD) proteins [21, 22]. Future work should include more comprehensive investigations of molecular mechanisms affected in trophoblast cells using RNA sequencing or proteomics data.

Recent research has shed light on the promising use of MSC-derived sEVs and other secreted factors, such as cytokines and miRNAs, for treating preeclampsia by promoting angiogenesis and regulating inflammation and oxidative stress [23–25]. Our study and others indicate that MSC-derived sEVs express the CD44 surface marker, which binds to FKBPL [20, 26]. MSC-conditioned media containing sEVs was shown to downregulate *Fkbpl* and increase CD44 mRNA expression in human umbilical vein endothelial cells. The same study demonstrated that MSC-derived conditioned media could enhance the migration of trophoblast cells, in both hypoxia and normoxia, relevant to preeclampsia [1]. Our findings further support these studies by demonstrating the internalization of MSC-derived sEVs by trophoblast cells. The observed cytoplasmic localization of fluorescently labeled sEVs aligns with previous reports and suggests potential trafficking to lysosomes for degradation or fusion with the endosomal membrane for content release, especially given the reduced intensity observed at 6 h [27]. Though a higher-resolution imaging system, such as super-resolution microscopy, could provide more precise counting of internalized sEVs, our use of confocal fluorescence microscopy is a commonly accepted method in the sEV field. Moreover, we noted nanotubule-like structures between trophoblasts containing fluorescently labeled sEVs, which may indicate a novel transport mechanism for sEVs in trophoblasts that has not been previously described. This discovery warrants further investigation to better understand sEV transport mechanisms and its potential implications for placental biology and the treatment of preeclampsia. While the use of hydrophobic dyes like PKH67 for fluorescent labeling poses a risk of forming fluorescent aggregates, leading to false-positive sEVs detection, we mitigated this through rigorous washing steps and analysis of the mean fluorescence intensity to normalize any unbound dye contributions. These measures ensured that our findings regarding sEVs uptake by trophoblasts were accurate and reliable. Establishing an optimal 6 h incubation period for subsequent experiments provides a foundation for future studies to evaluate the effects of sEVs on trophoblast cell migration.

Interestingly, partial *Fkbpl-*K/O seems to impede the uptake of sEVs at 2 and 4 h timepoints, potentially reducing their effect. This was corroborated by the lack of effect of sEVs on *Fkbpl-*K/O trophoblast proliferation, unlike in controls. MSC-derived sEVs were also unable to restore impaired trophoblast migration of *Fkbpl*-K/O trophoblast cell clones.

These results suggest that FKBPL could be critical in the MSC-sEV-mediated mechanism of action on trophoblast cells, likely through a receptor-dependent mechanism of internalization involving CD44 expressed on sEVs and FKBPL expressed on trophoblast cells. MSCs-derived sEVs are known to deliver bioactive cargo, including proteins, cytokines, and nucleic acids that mediate reparative functions in various pathologies, including placental disorders [28, 29]. Mechanistically, sEVs uptake by trophoblasts is dominated by endocytic pathways, clathrin- and caveolin-mediated endocytosis, macropinocytosis, and receptor-dependent internalization [30, 31]. Internalized vesicles traffic through endosomal compartments, arriving at early endosomes and multivesicular bodies, cargo is subsequently sorted for cytoplasmic release or lysosomal degradation [27]. Observations of PKH67-labeled sEVs localization in the cytoplasm within 4 h and the formation of nanotube-like inter-trophoblast connections suggest that both canonical endocytosis and potential membrane fusion or nanotube-mediated transfer contribute to sEVs delivery in trophoblast cells. Proteomics profiling of bone marrow-derived MSC-EVs has identified a shared core cargo exceeding 600 proteins, encompassing functional groups critical for cell proliferation, angiogenesis, Wnt signaling pathway, and extracellular matrix (ECM)/basement membrane modulation [32]. Notably, proteins involved in basement membrane integrity and ECM remodeling, including laminins, collagens, integrins, and protease regulators, were consistently identified [33], suggesting MSC-sEVs can directly influence the trophoblast microenvironment. Understanding these mechanisms could pave the way for developing new therapeutic strategies targeting trophoblast cell migration pathways in preeclampsia and other related conditions. Future work should include the impact on these proteins and pathways following partial *Fkbpl-*K/O. Although demonstrated previously in various in vivo models [23], the therapeutic effect of MSC-sEVs in the context of preeclampsia should be further explored in the rat reduced uterine perfusion pressure model of preeclampsia. Further studies could also test MSC-sEVs in the *Fkbpl*^*+*/−^ transgenic mouse model of preeclampsia to further elucidate the importance of FKBPL in the MSC-sEV-mediated mechanism of action [34]. In conclusion, CRISPR/Cas9-mediated gene editing offers a promising approach for modulating gene expression in cells, but effective delivery of the CRISPR/Cas9 complex remains a challenge. In this study, we evaluated the use of a novel and efficient MFP system for *Fkbpl-*targeting CRISPR pDNA delivery to investigate the role of *Fkbpl* in key placental cells in conditions relevant to a pregnancy complication, preeclampsia. To induce reduced expression of *Fkbpl* that was previously observed in women early in pregnancy who proceeded to develop preeclampsia [1], we employed various transfection approaches to deliver a CRISPR/Cas9 pDNA into trophoblast cells, resulting in the establishment of a number of genetically-edited *Fkbpl*-K/O cell lines, albeit full *Fkbpl* knockout was not viable. We then conducted downstream analyses to evaluate the impact of *Fkbpl* on trophoblast proliferation, migration and the therapeutic potential of MSC-derived sEVs on this process. In this study, we demonstrated that our MFP system improved gene editing in trophoblasts, which are typically difficult to transfect. These results underscore the potential of this technology for advancing biomedical research by enabling genetic manipulation that is gentle to cells and effective. Moreover, our results demonstrated that *Fkbpl* plays an important role in trophoblast cell proliferation and migration, and that in its absence, MSC-derived sEVs could not restore impaired trophoblast proliferation and migration. However, further studies are needed to elucidate the molecular mechanisms by which *Fkbpl* affects trophoblasts and MSC-derived sEVs mechanism of action. The findings of this research hold significant promise for advancing the development of potential therapeutics for the treatment of preeclampsia, a serious medical condition that was selected as a relevant real-world problem to address. Our MFP system could also be applied to other cell types to elucidate pathogenic mechanisms and evaluate therapies for other conditions. Future research should investigate the application of this technique to other cell types, such as primary trophoblast cells and pluripotent stem cells, and assess whether gene-editing efficiency in these cells is higher than that of traditional methods like lipofection or electroporation. Additionally, beyond cell proliferation and migration, further research is needed to elucidate whether the *Fkbpl* gene affects the functions of other placental cells, including immune cells.

## Supplementary information

Supplementary Material

Full Western blot images

## Data Availability

All data generated or analyzed during this study are available from the corresponding author upon reasonable request.
