# Supplementary material for: CRISPR/Cas9-mediated gene editing in trophoblast cells via mechanoporation for preeclampsia insight
Source: Cell Death Dis. 2025 Nov 24;17(1):61. doi: 10.1038/s41419-025-08200-z (PMC12827355; doi:10.1038/s41419-025-08200-z)
Supplement: Supplementary file 1 — Supplementary Material [file 41419_2025_8200_MOESM1_ESM.docx]

**Supplementary Data**

**Supplementary Table 1**. Participants' clinical characteristics.

| Clinical characteristic | First trimester  (n=10) | Preterm  (n=16) | Term  (n=10) | P-value |
| --- | --- | --- | --- | --- |
| Maternal age (y/o) | 26.5±6.17 | 29.63±7.26 | 33.40±3.63 | 0.0558 |
| Maternal BMI (Kg/m^2^) | 24.41±3.84 | 29.69±6.75 | 25.2±2.38 | 0.0306 |
| Parity | 0.7±0.8 | 1.1±1.2 | 0.7±0.9 | 0.669 |
| Gestational age (weeks) | 8.87±1.15 | 29.93±1.98 | 38.21±0.7 | <0.0001 |
| sBP (mmHg) |  | 122.3±10.19 | 120.4±6.3 | 0.612^t^ |
| dBP (mmHg) |  | 73.19±8.4 | 71.10±5.15 | 0.487^t^ |
| Birthweight (g) |  | 1496±393 | 3390±613 | <0.0001^t^ |

*Key*: BP: blood pressure, N/A: not applicable.

Data presented as mean±SEM, One-way ANOVA Kruskal-Wallis, ^t^ Unpaired t-test.

*sBP: Systolic Blood Pressure, dBP: Diastolic Blood Pressure.*

**Supplementary Table *2***. CRISPR FKBPL primer sequences.

| ***Primer*** | ***Sequence (5'→3')*** |
| --- | --- |
| *Fkbpl* forward (gRNA1) | CACCGGCAACCCCCCTTCGGTATA |
| *Fkbpl* reverse (gRNA1) | aaacTATACCGAAGGGGGGTTGCC |
| PCR primer forward | CCTCCTACCGAAACGCTTGA |
| PCR primer reverse | ATCTCACAGCACAGACCACAA |
| Sequencing primer forward | AACCCAAACTAGGCTCCTGC |
| Sequencing primer reverse | CCTGAATGACCACCTTCCCC |

**
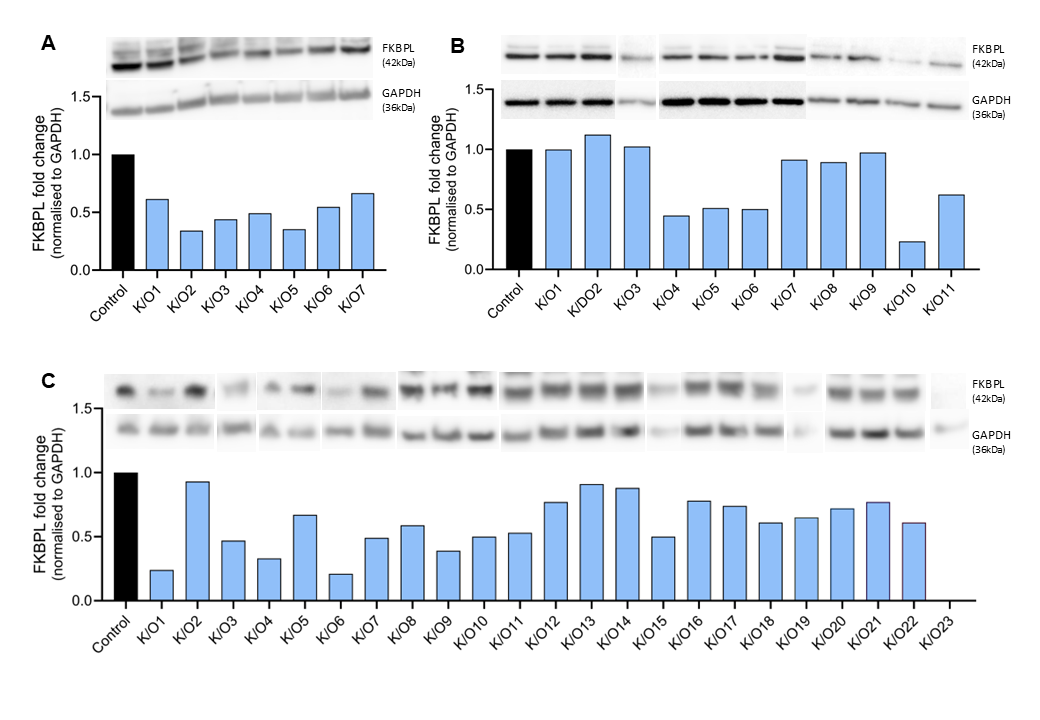
**

**Supplementary Figure 1** FKBPL protein analysis of knockdown clones following CRISPR/Cas9 transfection using lipofection, electroporation (EP) and microfiltroporation (MFP). Protein lysates from knockdown lines were subjected to western blotting. FKBPL band intensity was normalized to GAPDH signal for each sample and expressed as fold change compared to negative control. FKBPL protein expression was compared from knockdown/put clones generated by **(A)** lipofection, **(B)** EP and **(C)** MFP. *K/D: knockdown, K/O: knockout*.

**
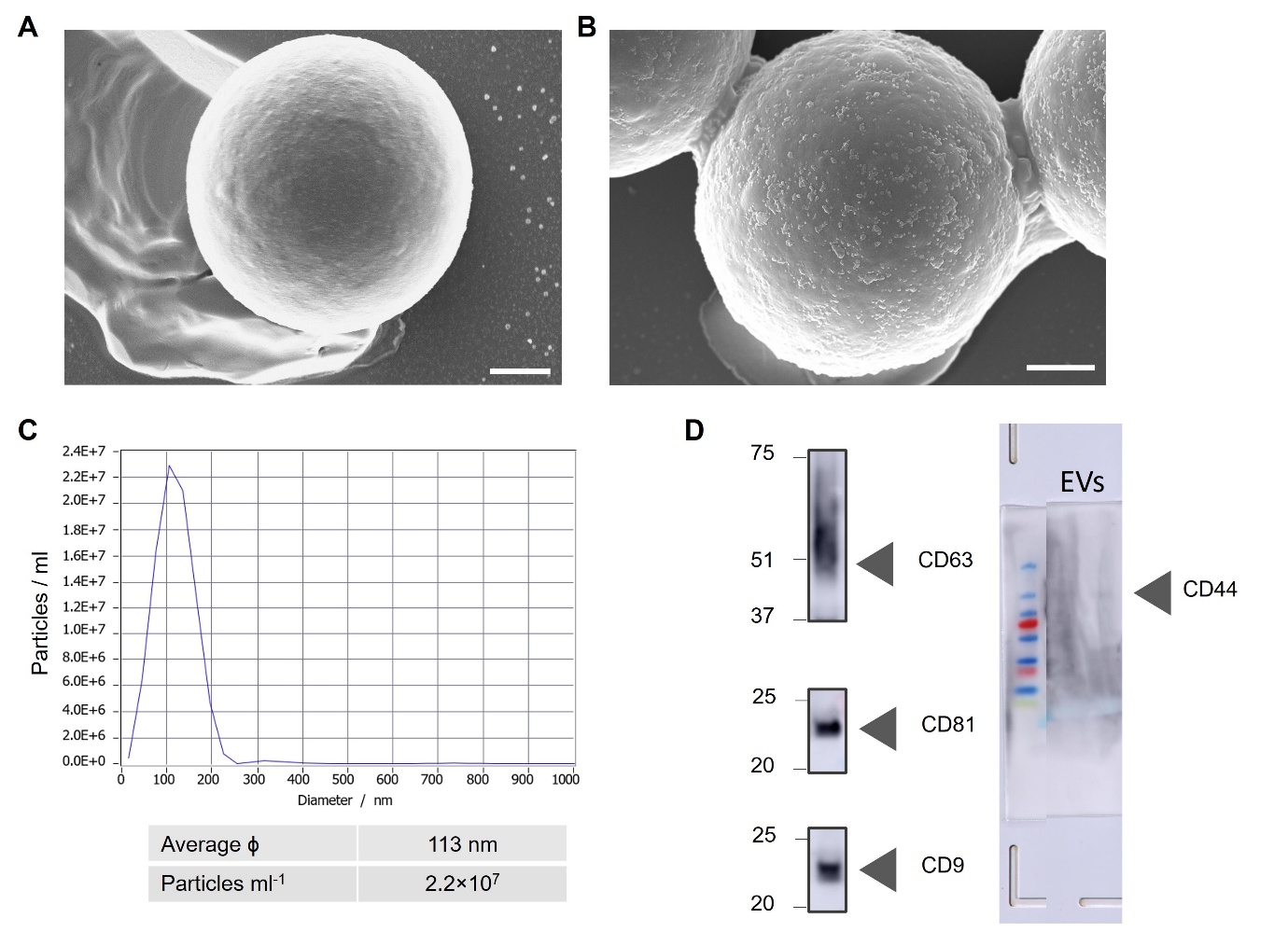
**

**Supplementary Figure 2** Characterization of MSC-derived sEVs. **(A, B)** These plans demonstrate the scanning electron microscopy images of polystyrene bead (10 µm) without coating **(A)** and beads coated with MSC-derived sEVs **(B)**. The scale bar represents 2 µm and the images were captured at the magnification of 5000×. **(C)** The NTA for the MSC-derived sEVs demonstrated the concentration and average size of the sEVs. **(D)** Western blot of MSC-derived sEV samples for CD63, CD81, CD9, and CD44.
