# Supplementary material for: CRISPR/Cas9-mediated gene editing in trophoblast cells via mechanoporation for preeclampsia insight
Source: Cell Death Dis. 2025 Nov 24;17(1):61. doi: 10.1038/s41419-025-08200-z (PMC12827355; doi:10.1038/s41419-025-08200-z)
Supplement: Supplementary file 2 — Full Western blot images [file 41419_2025_8200_MOESM2_ESM.pptx]

## Slide 1
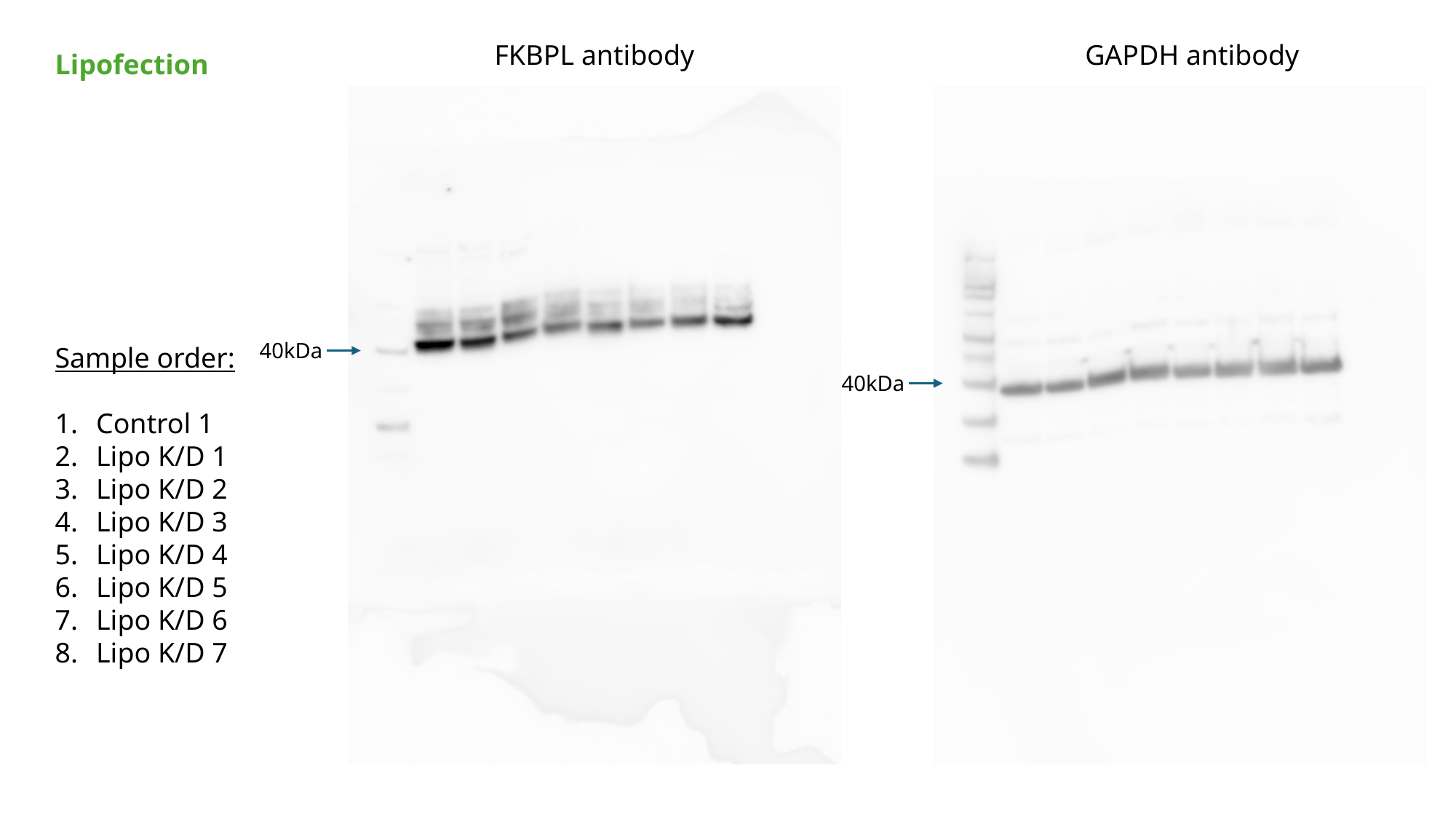

FKBPL antibody
GAPDH antibody
Lipofection
40kDa
Sample order:
Control 1
Lipo K/D 1
Lipo K/D 2
Lipo K/D 3
Lipo K/D 4
Lipo K/D 5
Lipo K/D 6
Lipo K/D 7
40kDa

## Slide 2
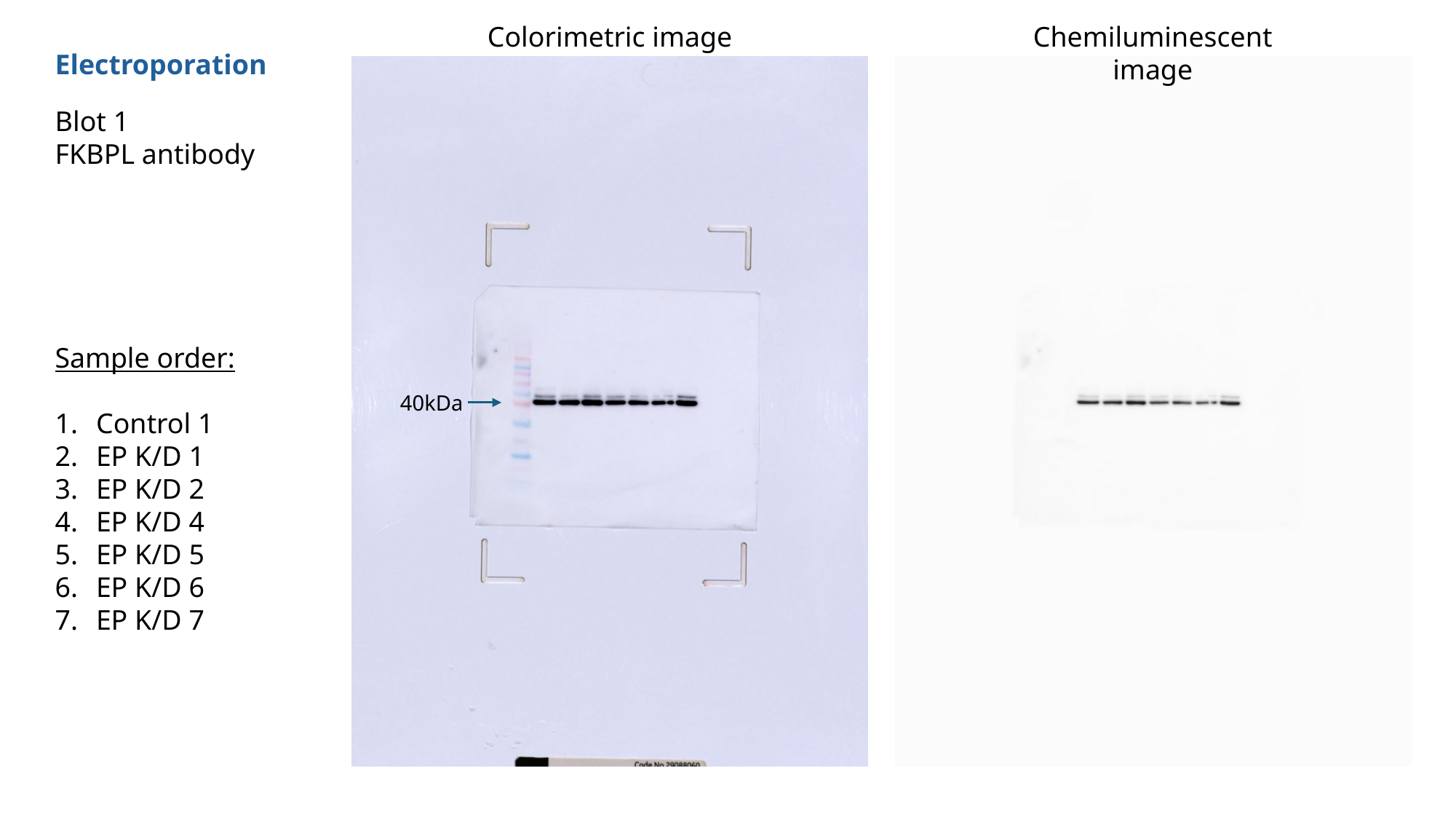

Colorimetric image
Chemiluminescent image
Electroporation
Blot 1
FKBPL antibody
Sample order:
Control 1
EP K/D 1
EP K/D 2
EP K/D 4
EP K/D 5
EP K/D 6
EP K/D 7
40kDa

## Slide 3
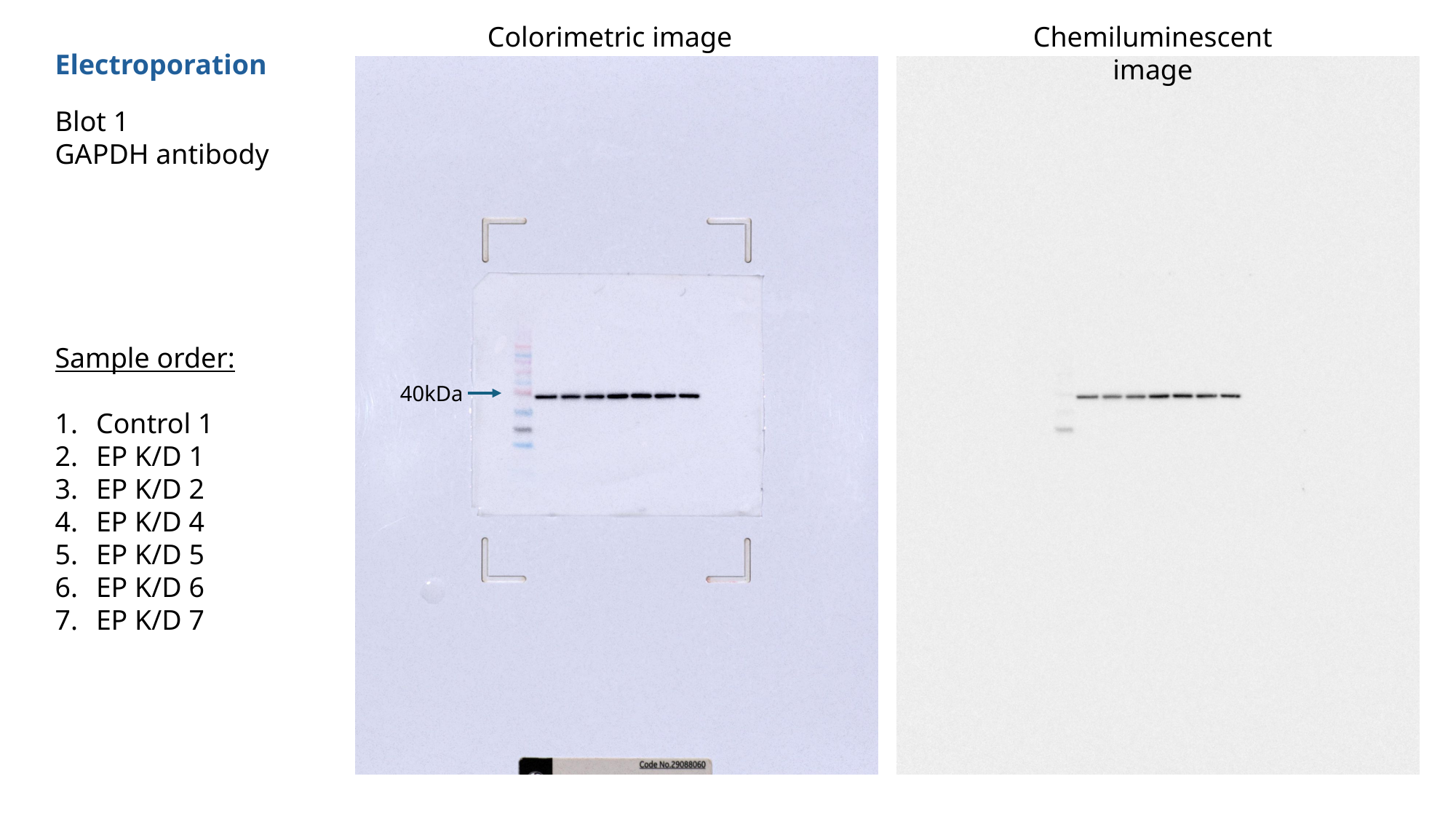

Colorimetric image
Chemiluminescent image
Electroporation
Blot 1
GAPDH antibody
Sample order:
Control 1
EP K/D 1
EP K/D 2
EP K/D 4
EP K/D 5
EP K/D 6
EP K/D 7
40kDa

## Slide 4
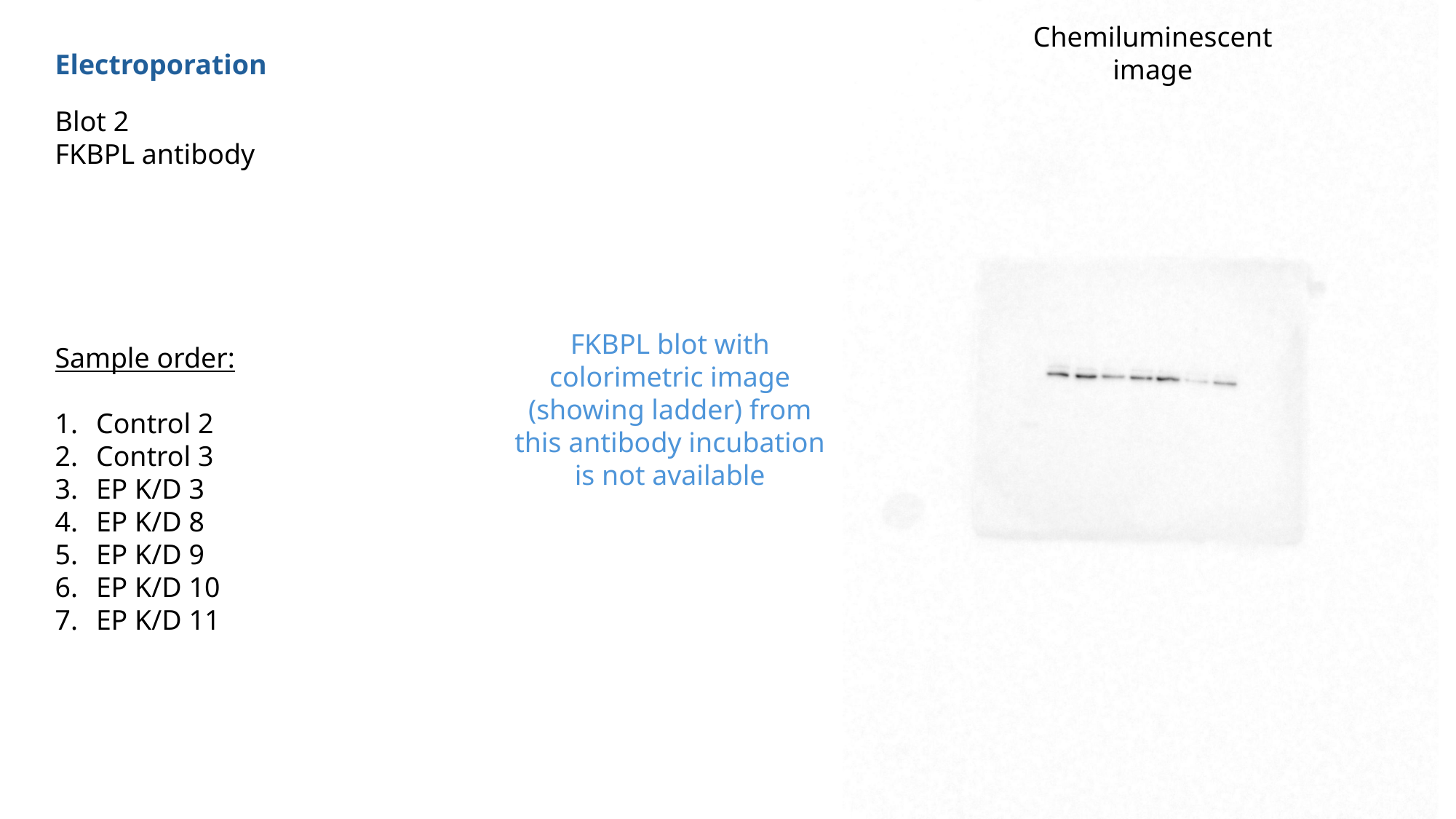

Chemiluminescent image
Electroporation
Blot 2
FKBPL antibody
FKBPL blot with colorimetric image (showing ladder) from this antibody incubation is not available
Sample order:
Control 2
Control 3
EP K/D 3
EP K/D 8
EP K/D 9
EP K/D 10
EP K/D 11

## Slide 5
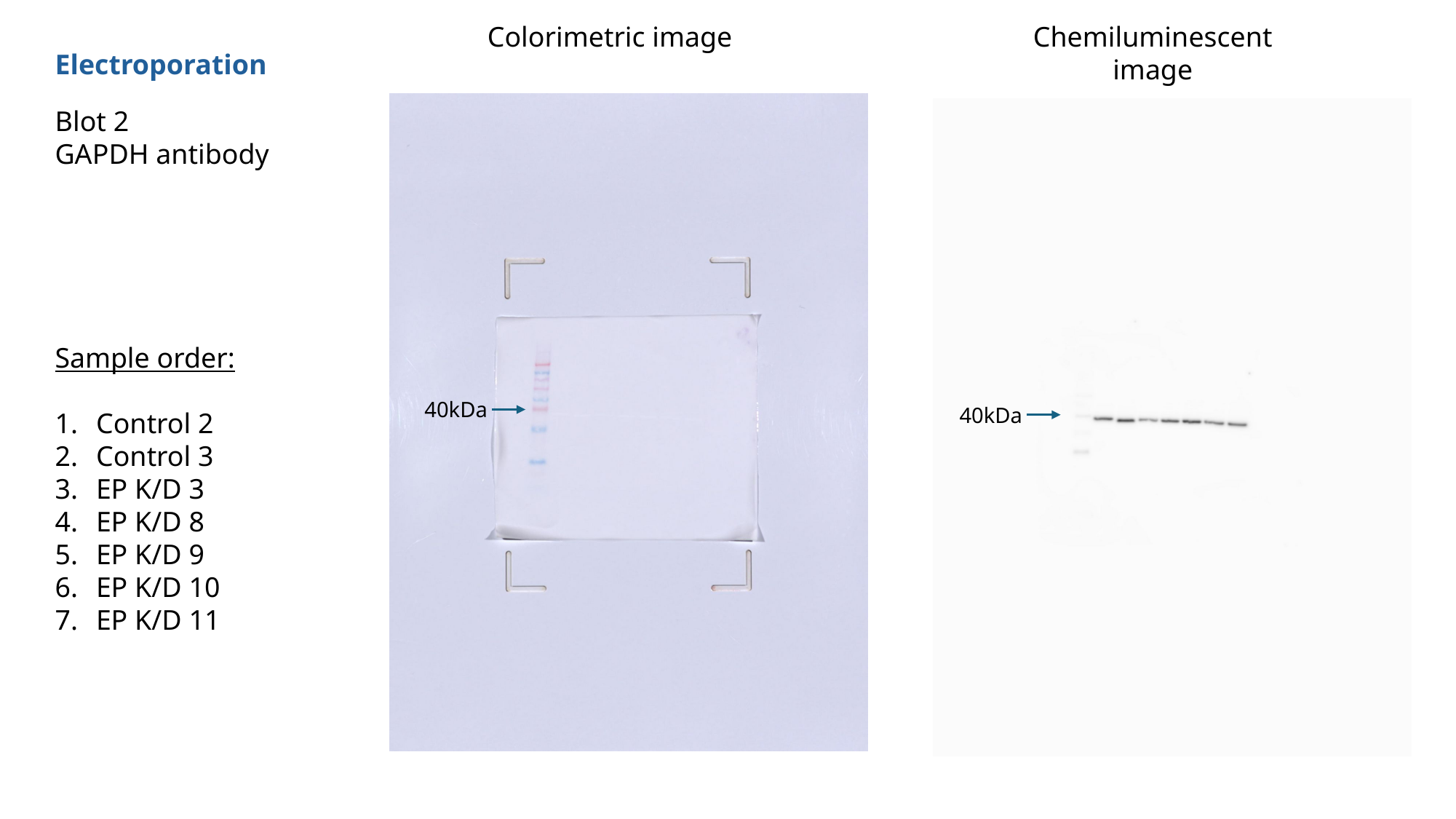

Colorimetric image
Chemiluminescent image
Electroporation
Blot 2
GAPDH antibody
Sample order:
Control 2
Control 3
EP K/D 3
EP K/D 8
EP K/D 9
EP K/D 10
EP K/D 11
40kDa
40kDa

## Slide 6
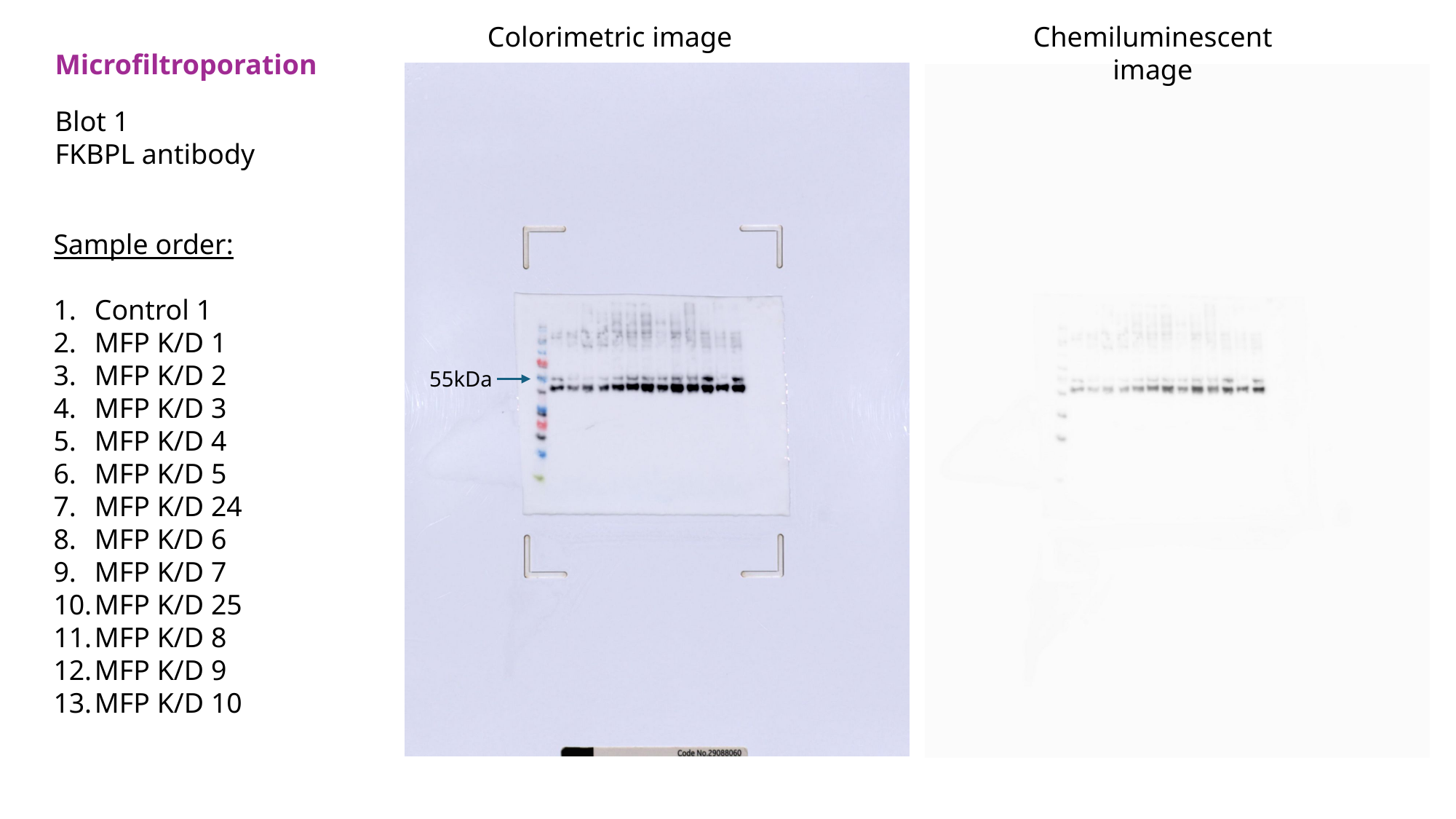

Colorimetric image
Chemiluminescent image
Microfiltroporation
Blot 1
FKBPL antibody
Sample order:
Control 1
MFP K/D 1
MFP K/D 2
MFP K/D 3
MFP K/D 4
MFP K/D 5
MFP K/D 24
MFP K/D 6
MFP K/D 7
MFP K/D 25
MFP K/D 8
MFP K/D 9
MFP K/D 10
55kDa

## Slide 7
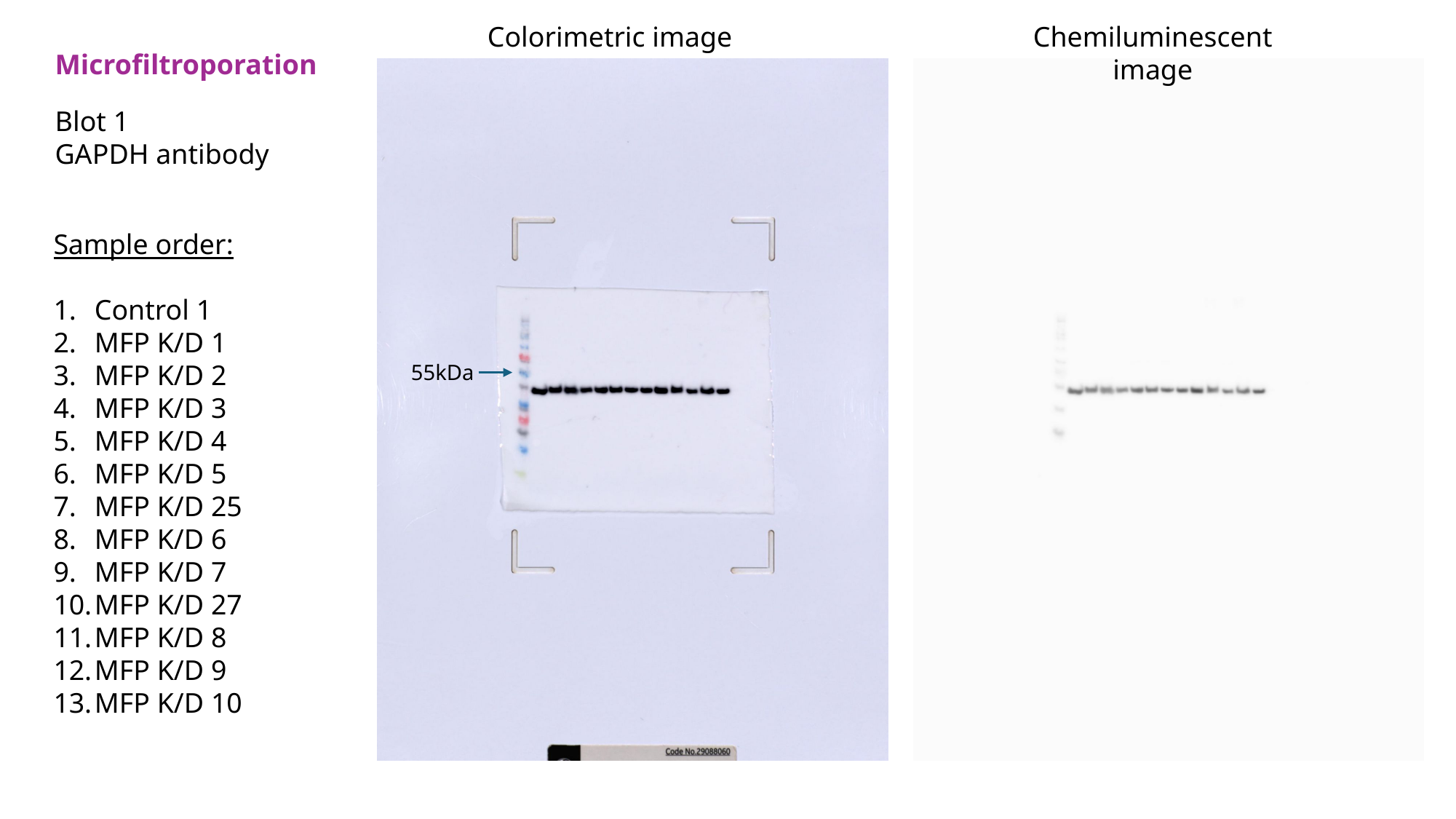

Colorimetric image
Chemiluminescent image
Microfiltroporation
Blot 1
GAPDH antibody
Sample order:
Control 1
MFP K/D 1
MFP K/D 2
MFP K/D 3
MFP K/D 4
MFP K/D 5
MFP K/D 25
MFP K/D 6
MFP K/D 7
MFP K/D 27
MFP K/D 8
MFP K/D 9
MFP K/D 10
55kDa

## Slide 8
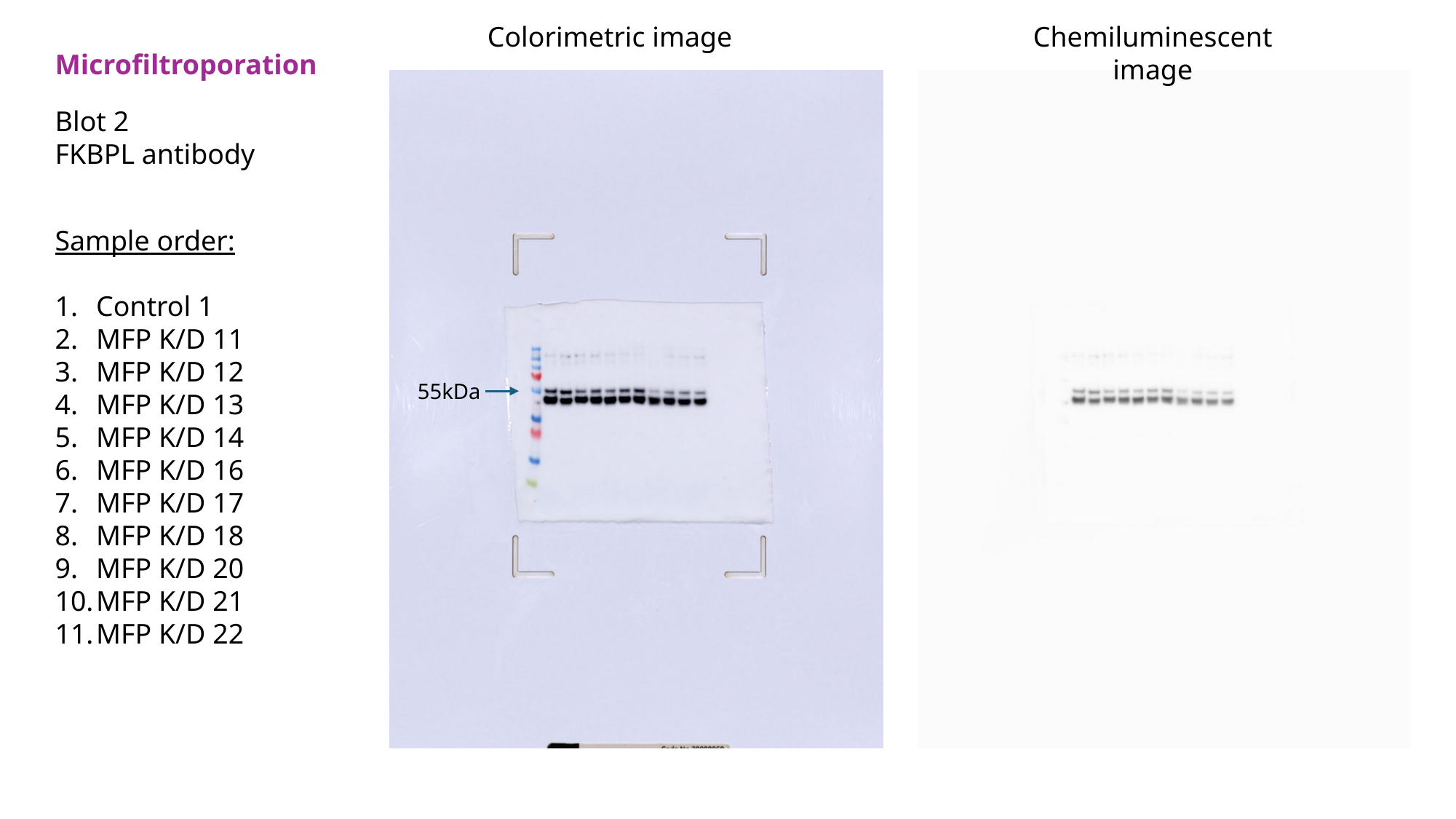

Colorimetric image
Chemiluminescent image
Microfiltroporation
Blot 2
FKBPL antibody
Sample order:
Control 1
MFP K/D 11
MFP K/D 12
MFP K/D 13
MFP K/D 14
MFP K/D 16
MFP K/D 17
MFP K/D 18
MFP K/D 20
MFP K/D 21
MFP K/D 22
55kDa

## Slide 9
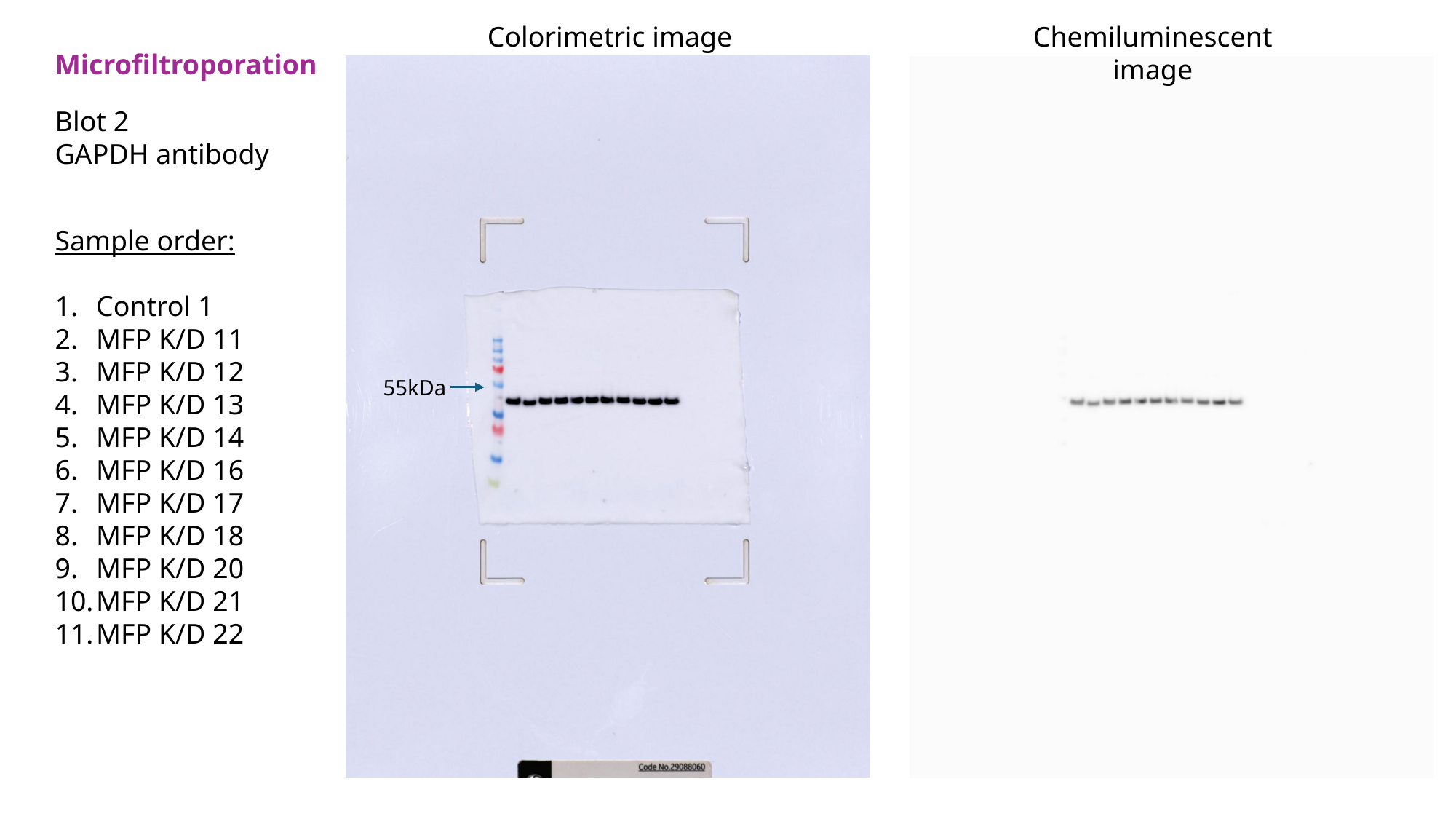

Colorimetric image
Chemiluminescent image
Microfiltroporation
Blot 2
GAPDH antibody
Sample order:
Control 1
MFP K/D 11
MFP K/D 12
MFP K/D 13
MFP K/D 14
MFP K/D 16
MFP K/D 17
MFP K/D 18
MFP K/D 20
MFP K/D 21
MFP K/D 22
55kDa

## Slide 10
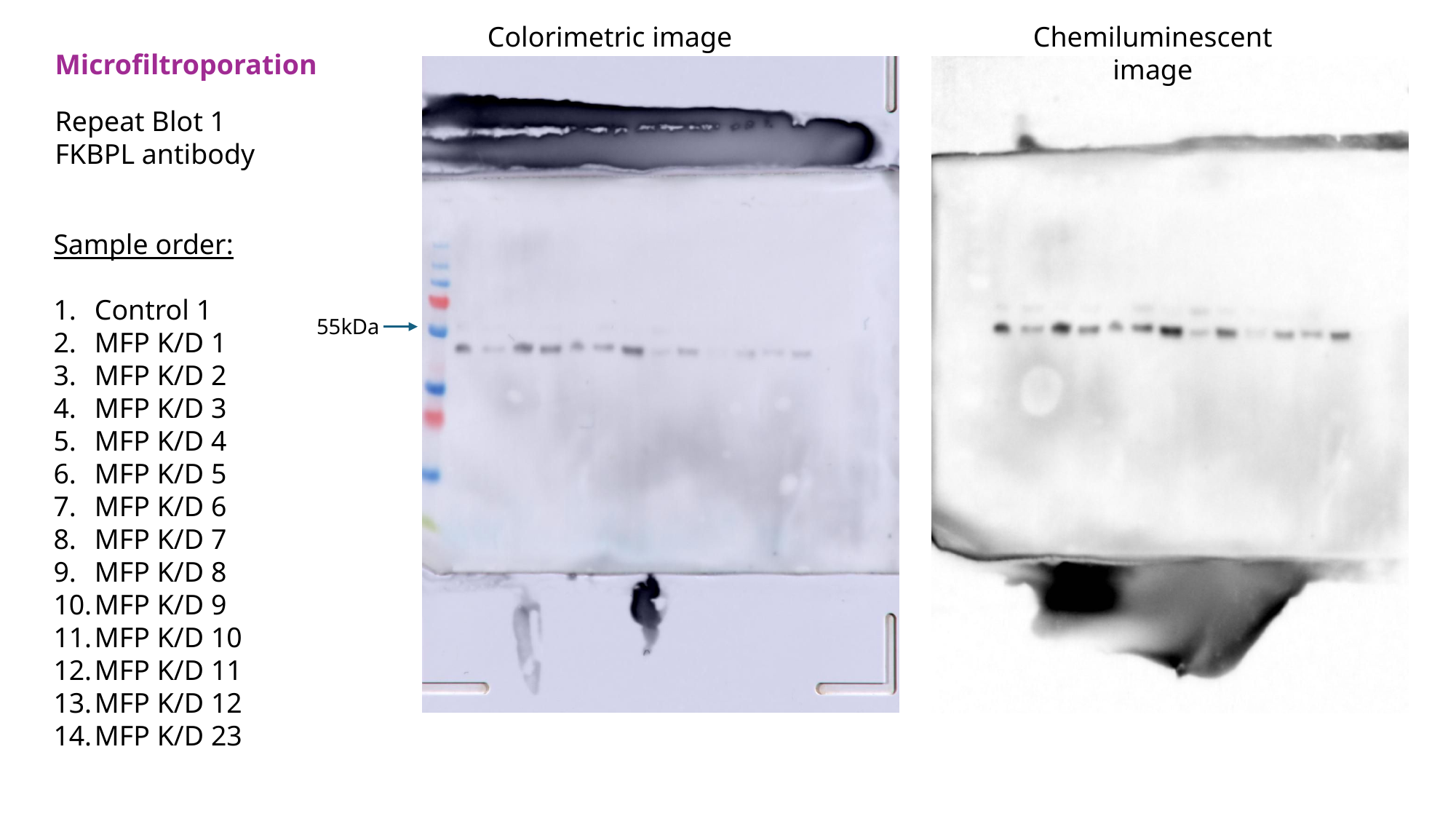

Colorimetric image
Chemiluminescent image
Microfiltroporation
Repeat Blot 1
FKBPL antibody
Sample order:
Control 1
MFP K/D 1
MFP K/D 2
MFP K/D 3
MFP K/D 4
MFP K/D 5
MFP K/D 6
MFP K/D 7
MFP K/D 8
MFP K/D 9
MFP K/D 10
MFP K/D 11
MFP K/D 12
MFP K/D 23
55kDa

## Slide 11
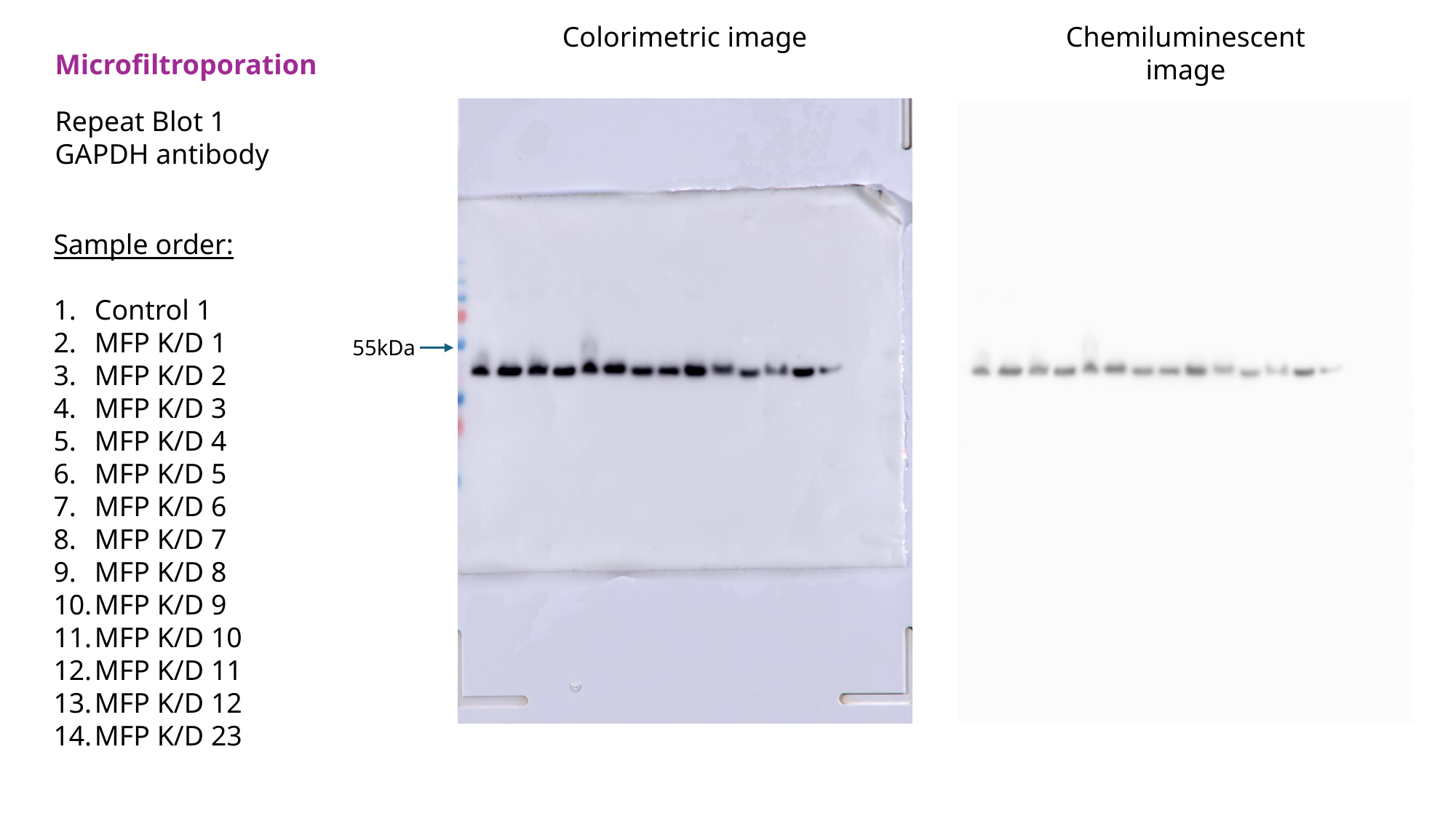

Colorimetric image
Chemiluminescent image
Microfiltroporation
Repeat Blot 1
GAPDH antibody
Sample order:
Control 1
MFP K/D 1
MFP K/D 2
MFP K/D 3
MFP K/D 4
MFP K/D 5
MFP K/D 6
MFP K/D 7
MFP K/D 8
MFP K/D 9
MFP K/D 10
MFP K/D 11
MFP K/D 12
MFP K/D 23
55kDa

## Slide 12
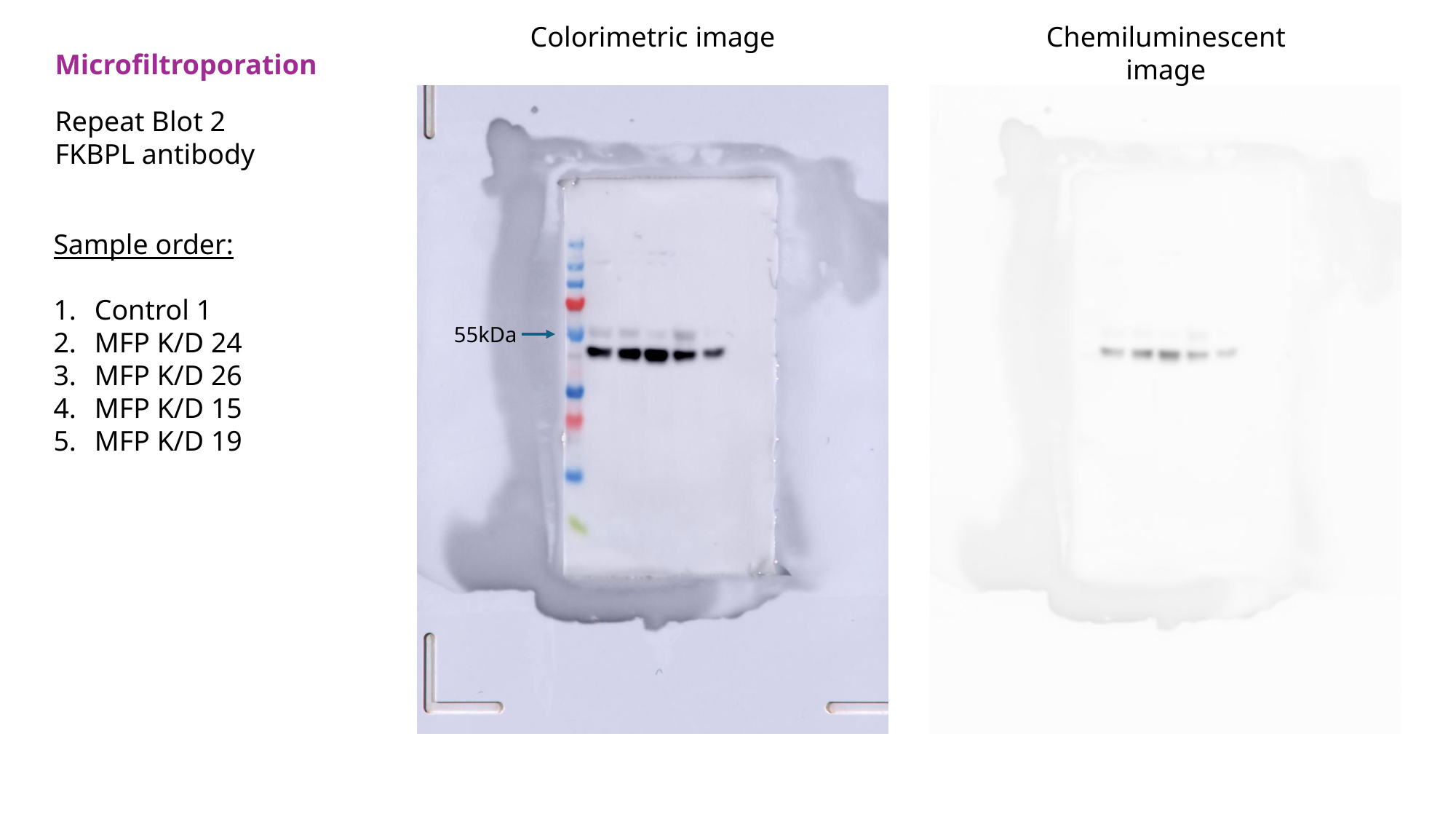

Colorimetric image
Chemiluminescent image
Microfiltroporation
Repeat Blot 2
FKBPL antibody
Sample order:
Control 1
MFP K/D 24
MFP K/D 26
MFP K/D 15
MFP K/D 19
55kDa

## Slide 13
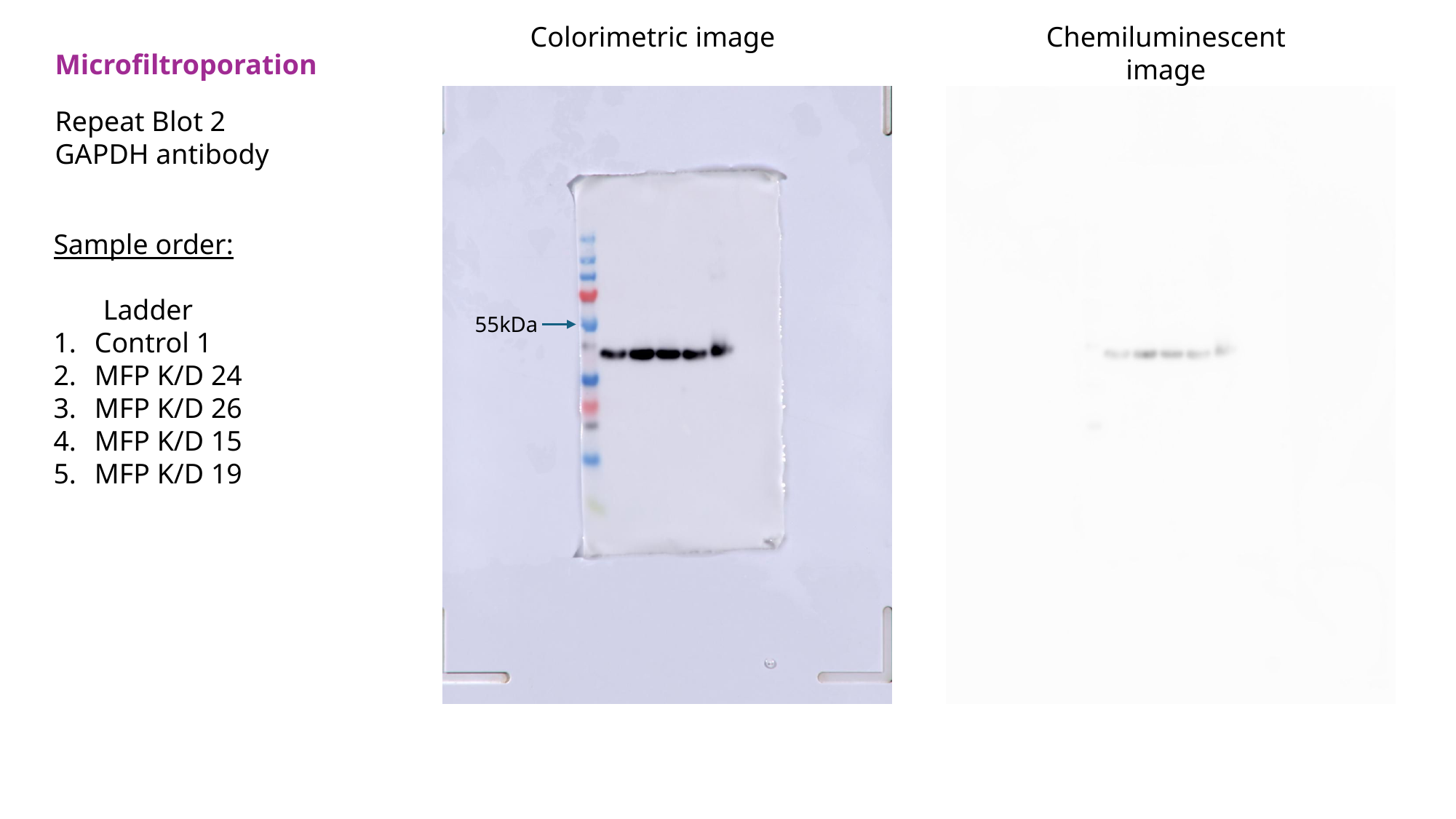

Colorimetric image
Chemiluminescent image
Microfiltroporation
Repeat Blot 2
GAPDH antibody
Sample order:
 Ladder
Control 1
MFP K/D 24
MFP K/D 26
MFP K/D 15
MFP K/D 19
55kDa
